# Development of a prognostic model of COVID-19 severity: a population-based cohort study in Iceland

**DOI:** 10.1186/s41512-022-00130-0

**Published:** 2022-09-08

**Authors:** Elias Eythorsson, Valgerdur Bjarnadottir, Hrafnhildur Linnet Runolfsdottir, Dadi Helgason, Ragnar Freyr Ingvarsson, Helgi K. Bjornsson, Lovisa Bjork Olafsdottir, Solveig Bjarnadottir, Arnar Snaer Agustsson, Kristin Oskarsdottir, Hrafn Hliddal Thorvaldsson, Gudrun Kristjansdottir, Aron Hjalti Bjornsson, Arna R. Emilsdottir, Brynja Armannsdottir, Olafur Gudlaugsson, Sif Hansdottir, Magnus Gottfredsson, Agnar Bjarnason, Martin I. Sigurdsson, Olafur S. Indridason, Runolfur Palsson

**Affiliations:** 1 Landspitali–The National University Hospital of Iceland, Reykjavik, Iceland; 2 Faculty of Medicine, School of Health Sciences, University of Iceland, Reykjavik, Iceland

**Keywords:** SARS-CoV-2, COVID-19, Clinical decision rules, Prognostic model, Prediction model

## Abstract

**Background:**

The severity of SARS-CoV-2 infection varies from asymptomatic state to severe respiratory failure and the clinical course is difficult to predict. The aim of the study was to develop a prognostic model to predict the severity of COVID-19 in unvaccinated adults at the time of diagnosis.

**Methods:**

All SARS-CoV-2-positive adults in Iceland were prospectively enrolled into a telehealth service at diagnosis. A multivariable proportional-odds logistic regression model was derived from information obtained during the enrollment interview of those diagnosed between February 27 and December 31, 2020 who met the inclusion criteria. Outcomes were defined on an ordinal scale: (1) no need for escalation of care during follow-up; (2) need for urgent care visit; (3) hospitalization; and (4) admission to intensive care unit (ICU) or death. Missing data were multiply imputed using chained equations and the model was internally validated using bootstrapping techniques. Decision curve analysis was performed.

**Results:**

The prognostic model was derived from 4756 SARS-CoV-2-positive persons. In total, 375 (7.9%) only required urgent care visits, 188 (4.0%) were hospitalized and 50 (1.1%) were either admitted to ICU or died due to complications of COVID-19. The model included age, sex, body mass index (BMI), current smoking, underlying conditions, and symptoms and clinical severity score at enrollment. On internal validation, the optimism-corrected Nagelkerke’s *R*^2^ was 23.4% (95%CI, 22.7–24.2), the C-statistic was 0.793 (95%CI, 0.789-0.797) and the calibration slope was 0.97 (95%CI, 0.96–0.98). Outcome-specific indices were for urgent care visit or worse (calibration intercept -0.04 [95%CI, -0.06 to -0.02], *E*_max_ 0.014 [95%CI, 0.008–0.020]), hospitalization or worse (calibration intercept -0.06 [95%CI, -0.12 to -0.03], *E*_max_ 0.018 [95%CI, 0.010–0.027]), and ICU admission or death (calibration intercept -0.10 [95%CI, -0.15 to -0.04] and *E*_max_ 0.027 [95%CI, 0.013–0.041]).

**Conclusion:**

Our prognostic model can accurately predict the later need for urgent outpatient evaluation, hospitalization, and ICU admission and death among unvaccinated SARS-CoV-2-positive adults in the general population at the time of diagnosis, using information obtained by telephone interview.

**Supplementary Information:**

The online version contains supplementary material available at 10.1186/s41512-022-00130-0.

## Introduction

Coronavirus disease 2019 (COVID-19), caused by the severe acute respiratory syndrome-coronavirus-2 (SARS-CoV-2), was first described in Wuhan, China, in December 2019 and was declared a pandemic on March 11, 2020 [1]. The severity of COVID-19 ranges from asymptomatic infection to severe respiratory failure and death. While early reports suggested that most infections were severe, later studies found that 81% of those who were symptomatic had mild disease, 14% had severe disease, and 5% developed critical illness [2]. The infection fatality rate has been estimated to be between 0.26 and 0.66% [3, 4].

Since the first diagnosed case of COVID-19 in Iceland on February 27, 2020 and until December 31, 2020, a total of 6,126 persons tested SARS-CoV-2-positive by quantitative reverse-transcriptase polymerase chain reaction (qPCR). Broad access to qPCR testing was introduced early in the pandemic for both symptomatic and asymptomatic persons. All individuals who tested positive were enrolled into a telehealth service provided by the COVID-19 Outpatient Clinic of Landspitali–The National University Hospital of Iceland (LUH) [5]. The entire cohort of SARS-CoV-2-positive cases was prospectively followed from the date of diagnosis. A national seroprevalence study found that a large proportion (56%) of seropositive individuals had been identified by qPCR testing during the first wave of the pandemic in Iceland [6].

While extensive testing and follow-up is desirable for contact tracing and isolation of infected people, large numbers of cases can easily overwhelm the capacity of the healthcare system for provision of clinical care. Early risk stratification offers opportunities for triaging SARS-CoV-2-positive persons to appropriate levels of monitoring and intervention. A living systematic review and meta-analysis of prediction models for COVID-19 has identified 107 models that predict the prognosis of COVID-19 [7]. Of those models, only one predicting the prognosis of COVID-19 among individuals in the general population (QCOVID) was judged to be at low risk of bias [8]. QCOVID predicts the 90-day risk of being infected with SARS-CoV-2 and subsequently hospitalized or dying in the UK. It has since been externally validated and has incorporated two-dose vaccination status as a predictor [9, 10]. However, QCOVID does not predict the need for urgent outpatient evaluation for COVID-19, and it is unclear how certain predictors can be implemented for use in other countries.

The aim of this study was to develop a multivariable model to predict the risk of urgent outpatient evaluation, hospitalization, and intensive care unit (ICU) admission or death among unvaccinated SARS-CoV-2-positive adults at the time of diagnosis in order to assist clinicians in prioritizing infected individuals for clinical monitoring and early therapeutic intervention.

## Methods

### Ethical approval

The study was approved by the National Bioethics Committee of Iceland (VSN 20-078).

### Study population

**Fig. 1:**
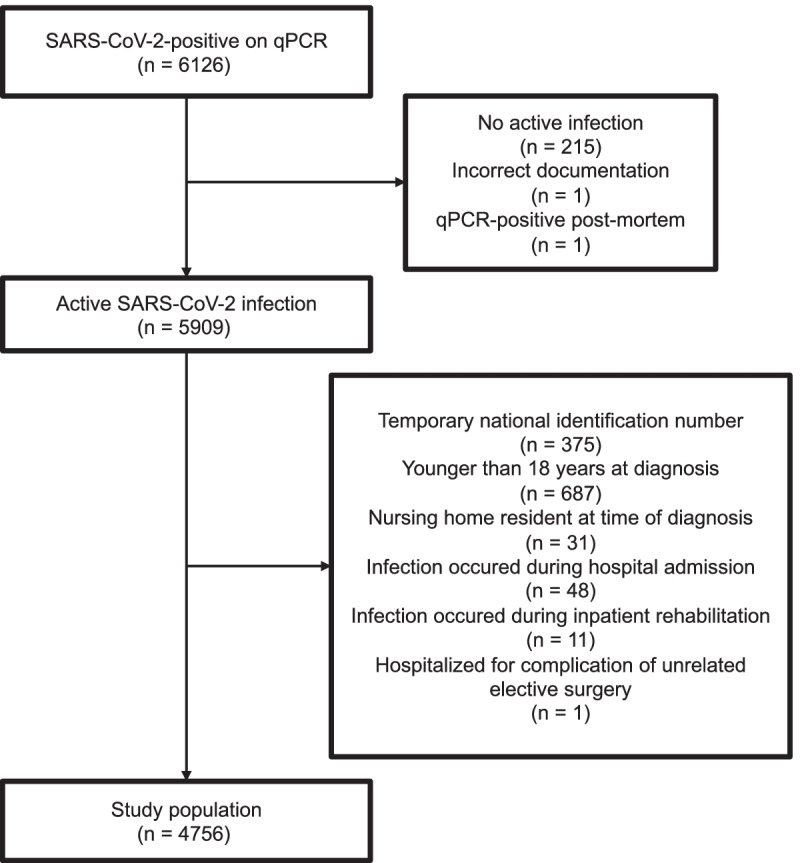
Flow diagram of the study cohort

The study population included all persons who tested positive for SARS-CoV-2 by qPCR in Iceland between February 27 and December 31, 2020 with the exception of those who met the exclusion criteria. During this period, no person had been vaccinated for SARS-CoV-2. Three national testing programs were implemented during the study period; targeted testing based on clinical suspicion (from February 1), open invitation population screening (from March 13) and mandatory screening at the border (from June 15). Due to the oppressive nature of enforced quarantine, those who tested positive at border screening underwent antibody testing, a repeat qPCR test of a nasopharyngeal swab and an assessment of symptoms and exposure, and the presence of an active infection was determined based on these data. All persons who were SARS-CoV-2-positive and considered to have an active infection were enrolled into the telehealth service of the LUH COVID-19 Outpatient Clinic until uneventful discontinuation of follow-up care, hospital discharge or death from COVID-19. The derivation cohort excluded children younger than 18 years of age, individuals without an Icelandic national identification number, and those living in a nursing home or who were admitted to hospital at the time of diagnosis (Fig. 1).

### The COVID-19 Outpatient Clinic

The COVID-19 Outpatient Clinic coordinated the outpatient care of all SARS-CoV-2-positive persons in Iceland as described previously [5]. Data regarding underlying conditions, medication use, clinical symptoms and severity of the infection were prospectively recorded using a standardized data entry form starting on March 17. During each interview, the presence of 19 specific symptoms was documented. Patients were evaluated and assigned a clinical severity score by the interviewing physician or nurse, based on the combination of their symptoms and the clinical judgment of the healthcare provider. The clinical severity score was generated by infectious disease consultants at LUH at the beginning of the pandemic: (1) low severity, defined as having mild or no symptoms; (2) moderate severity, defined as mild dyspnea, cough, or fever for less than 5 days; and (3) high severity, defined as severe dyspnea, worsening cough, and high or persistent fever for 5 days or longer. Patients with alarming symptoms were brought to the COVID-19 Outpatient Clinic for in-person evaluation. Patients were discharged from telehealth follow-up when at least 14 days had passed from qPCR-based diagnosis and at least seven days from the resolution of symptoms.

### Data sources

In addition to the prospectively collected information that was obtained through telehealth interviews, data were retrieved from several population-based registries in Iceland. All International Classification of Diseases, 10th Revision (ICD-10) diagnosis codes recorded ≥ 14 days prior to the persons’ first positive qPCR test for SARS-CoV-2 were obtained from three registries: LUH patient registry (from 2009), the Register of Primary Health Care Contacts (from 2004) and the Register of Contacts with Medical Specialists in Private Practice (from 2010). Data on all filled drug prescriptions were collected for each individual from the Prescription Medicines Register for the period ranging from 395 days (13 months) to 14 days before the first positive qPCR test for SARS-CoV-2. Finally, all measurements of serum creatinine between January 2010 and until 14 days before the individual’s first positive qPCR test were extracted from a central laboratory database. Estimated glomerular filtration rate (eGFR) was calculated from serum creatinine using the Chronic Kidney Disease Epidemiology Collaboration (CKD-EPI) equation [11].

### Prognostic model development

A proportional-odds logistic model was used and outcomes were defined on an ordinal scale, ranging from the least to the most severe: (1) absence of clinical deterioration requiring in-person evaluation or hospitalization for the duration of telehealth care; (2) clinical deterioration requiring urgent in-person evaluation at the LUH COVID-19 Outpatient Clinic, but not subsequent hospitalization; (3) hospitalization; and (4) admission to ICU or death. A formal minimum sample size calculation was performed to justify the number of candidate predictor variables using a modification of the four-step approach for binary models recommended by Riley et al. [12, 13]. Assuming 18 candidate predictor variables, the result was the following: (1) a minimum of 2796 persons would be required to estimate the proportion experiencing each outcome with a targeted margin of error of 1% or less; (2) a minimum of 2269 persons would be required to target a mean absolute prediction error of 2% or less for each of the outcomes; (3) assuming a conservative Cox-Snell *R*^2^ of only 0.09 for the binary prediction of any outcome, a minimum of 3506 persons would be required for the desired shrinkage of the predictor effects to be 5% or smaller; and finally (4) to target 2% optimism or less in the model‘s apparent Nagelkerke‘s *R*^2^, a minimum of 1584 persons would be required. The estimated minimum required sample size was therefore 3506. Predictor variables were derived from the prospectively recorded data obtained during the enrollment interview into the telehealth service. Age was modelled as a non-linear variable using a restricted cubic spline with knots placed at the 0.05, 0.35, 0.65, and 0.95 percentiles. Body mass index (BMI) was included as a linear variable. All other predictor variables were dichotomous. All decisions regarding predictors and model development were made prior to examining the outcome data and were based on the existing literature and clinical expertise of the study authors. No statistical selection procedures or model comparisons were employed. Two candidate predictor variables (chronic kidney disease and clinical score = high severity) were not included in the final model due to small sample sizes. The 16 predictor variables included in the prognostic model are shown in Table 1. Missing data were imputed 2000 times using multiple imputation with chained equations (MICE) and predictive mean matching carried out using the *aregImpute* function [14] Proportional odds assumptions and missingness mechanisms were explored [15]. Penalized maximum likelihood estimation was considered over a range of penalty factors (0, 0.5, and 1) for both linear and non-linear terms and values chosen to maximize the corrected Akaike information criterion. Detailed definitions of all variables included in the prognostic model and imputation procedure are provided in Supplementary Table 1.

**Table 1:** Variables included in the prognostic model are shown for the derivation cohort

Predictor	Derivation cohort *n* = 4756 Median and interquartile range for continuous or *n* and % for categorical variables (*n* missing and %)
Age, years	40, 28–54 (0, 0%)
Sex, male	2455, 51.6% (0, 0%)
Body mass index	26.0, 23.1–29.45 (1,441, 30.3%)
Current smoking	395, 9.1% (494, 8.5%)
Diabetes	136, 3.0% (242, 5.1%)
Hypertension	569, 12.6% (225, 0.7%)
Heart disease	282, 6.2% (235, 4.9%)
Chronic kidney disease	25, 0.6% (246, 5.2%)
Pulmonary disease	245, 5.4% (239, 5.0%)
Cancer	114, 2.5% (243, 5.1%)
Flu-like symptoms	3781, 80.8% (77, 2.2%)
Upper respiratory symptoms	2727, 59.5% (169, 3.6%)
Lower respiratory symptoms	1206, 26.6% (226, 4.8%)
Gastrointestinal symptoms	1018, 22.6% (244, 5.1%)
Clinical score = moderate or high severity	470, 10.6% (306, 6.4%)
Telehealth only	4143, 87.1% (0, 0%)
Urgent care visit	375, 7.9% (0, 0%)
Hospitalization	188, 4.0% (0, 0%)
Intensive care unit admission or death	50, 1.1% (0, 0%)

Continuous variables are summarized as medians and interquartile ranges (IQR). The number of cases behind each categorical variable are presented along with the percentage. For each of the variables, the number and proportion of cases with missing data are displayed within parenthesis. Two candidate predictor variables (chronic kidney disease [*n* = 25] and clinical score = high severity [*n* = 74]) were not included in the final model due to small sample sizes

Internal validation was performed by calculating and presenting optimism-corrected indices of fit, discrimination and calibration. The overall model fit was quantified with Nagelkerke‘s *R*^2^ and discrimination was quantified using the C-statistic. Calibration was assessed by visual examination of calibration plots and from the overall calibration slope and several outcome specific calibration indices (calibration intercept, Brier score, and *E*_max_) [16]. Optimism-corrected confidence intervals (CI) that incorporated both imputation and bootstrap variability were constructed by 2000 iterations of the following procedure: (1) sequential selection of each of the 2000 imputed datasets; (2) fit of the proportional-odds model to the selected dataset; (3) refit of the model on 200 bootstrap resamples with replacement of the selected dataset to calculate the optimism using the .632 estimator; (4) the extraction of the optimism-corrected indices and calibration curve [14, 17]. The lower and upper limits of the CI were defined as the 2.5 and 97.5 percentiles of the resulting distributions. The sensitivity, specificity, positive predictive value (PPV) and negative predictive value (NPV) of the model were reported for each outcome over a range of probabilistic thresholds. Decision curve analysis was performed to quantify the standardized net benefit of the prognostic model over a range of plausible probability thresholds for use in two clinical scenarios: (1) to aid in determining whether an individual‘s risk of clinical deterioration requiring an urgent care visit or worse is sufficiently small for omission from the telehealth service; and (2) to aid in deciding whether an individual’s risk of hospitalization or worse is sufficiently large to recommend more intensive follow-up or therapeutic intervention [18]. The decision curve analyses are described in more detail in the Supplementary Methods. In addition, the potential impact of implementing the model in the COVID-19 Outpatient Clinic was explored by determining the number of interviews that would have been prevented if those who had a predicted risk of requiring an urgent care visit or worse below a given threshold, received only two interviews (at enrollment and discharge) instead of the observed number of interviews. The prognostic model was reported according to the Transparent Reporting of a multivariable prediction model for Individual Prognosis or Diagnosis (TRIPOD) guidelines [19].

All statistics were performed in R version 3.6.3 [20]. using the *tidyverse* [21] package for data manipulation. The *cowplot* package [22] was used to create multipanel figures and *tableone* [23] was used to create summary tables. All statistical code is available at https://osf.io/t2bp8/.

## Results

### Study population

Of the 175,243 persons in Iceland who were tested for SARS-CoV-2 using qPCR during the study period, 6126 were positive. After applying exclusion criteria, 4756 persons were included (Fig. 1). The mean age was 42 years (median 40 years, IQR, 28–54) and the proportion of males was 51.6% (Table 1). In total, 4143 (87%) individuals never required in-person evaluation during telehealth follow-up, 375 (7.9%) required only urgent outpatient evaluation, 188 (4.0%) required admission to hospital but no further escalation of care, and 50 (1.1%) either required admission to ICU or died due to complications of COVID-19. The median time from the first positive qPCR test to telehealth enrollment was 0 days (IQR, 0–1, range 0–4), and the median follow-up time was 15 days (IQR, 14–16, range, 7–67). No person was lost to follow-up.

### Prognostic model performance

**Fig. 2:**
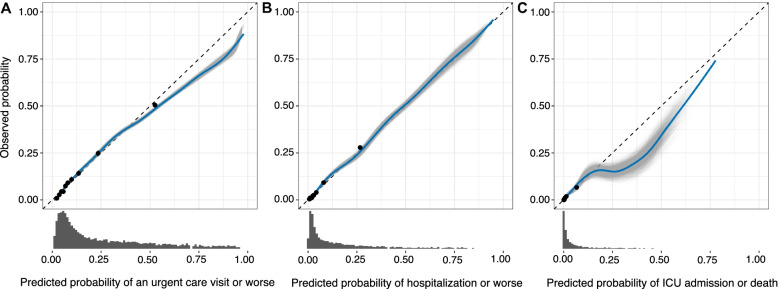
Optimism-corrected calibration curves of the prognostic model illustrate the relationship between the observed and predicted probability of urgent care visit or worse (**A**), hospitalization or worse (**B**) and admission to intensive care unit or death (**C**). The sample distribution of predicted probabilities is presented as marginal histograms. The sample is divided into 10 equally large groups of predicted probability and the mean observed probability of each group depicted as a black dot and point range centered at the mean predicted probability of the group. The weighted scatterplot smoothing (LOWESS) relationship between the observed and predicted probabilities of bootstrap resamples with replacement from 2000 imputed datasets are shown as individual thin gray lines with the mean relationship shown as a blue line. These are compared to the dashed black line, reflecting a perfect relationship between observed and predicted probabilities

The Akaike information criterion was maximized at a penalty factor of 0 for both linear and non-linear terms and the model was therefore estimated using standard maximum likelihood. Visual examination of diagnostic figures suggested that the proportional odds and missingness assumptions were met (Supplementary Figures 1, 2, 3, 4, 5, 6, 7, 8 and 9). In our cohort, the median predicted probability of urgent care visit or worse was 7.2% (IQR, 4.6–13.2, with a 97.5 percentile of 62.4%), of hospitalization or worse was 2.1% (IQR, 1.3–4.1, with a 97.5 percentile of 31.7%), and the median predicted probability of ICU admission or death was 0.34% (IQR, 0.21–0.66, with a 97.5 percentile of 6.7%). On internal validation, the optimism-corrected Nagelkerke‘s *R*^2^ was 23.4% (95%CI, 22.7–24.2), the C-statistic was 0.793 (95%CI, 0.789–0.797) and the calibration slope was 0.97 (95%CI, 0.96–0.98) (Table 2). Outcome-specific optimism-corrected metrics were for urgent care visit or worse (calibration intercept -0.04 [95%CI, -0.06 to -0.02], *E*_max_ 0.014 [95%CI, 0.008–0.020], and Brier score 0.092 [95%CI, 0.091–0.093]), for hospitalization or worse (calibration intercept -0.06 [95%CI, -0.12 to -0.03], Emax 0.018 [95%CI, 0.010–0.027], and Brier score 0.039 [95%CI, 0.038–0.039]), and for ICU admission or death (calibration intercept -0.10 [95%CI, -0.15 to -0.04] and *E*_max_ 0.027 [95%CI, 0.013–0.041], and Brier score 0.010 [95%CI, 0.010−0.010]) (Table 2, Supplementary Table 3). Visual examination of the optimism-corrected calibration plots revealed excellent calibration with a tendency to overestimate risk at the extremes of the predicted risk (Fig. 2).

**Table 2:** Optimism-corrected calibration and discrimination indices of the prognostic model for each of the outcomes. The 95% bootstrapped confidence intervals are presented within parenthesis

Indexes	Urgent care visit	Hospitalization	Intensive care unit admission or death
C-statistic	0.793 (0.789 to 0.797)		
Negalkerke’s R^2^	0.234 (0.227 to 0.242)		
Calibration intercept	-0.043 (-0.064 to -0.023)	-0.063 (-0.094 to -0.033)	-0.098 (-0.155 to -0.042)
Calibration slope	0.973 (0.963 to 0.983)		
Brier score	0.092 (0.091 to 0.093)	0.039 (0.038 to 0.039)	0.010 (0.010 to 0.010)
*E*_max_	0.014 (0.008 to 0.020)	0.018 (0.010 to 0.027)	0.027 (0.013 to 0.041)

*E*_max_ is the maximum absolute difference between the predicted probabilities of the prognostic model and the weighted scatterplot smoothing (LOWESS) calibrated probability

The lower limit of the 95% CI for the NPV of requiring an urgent care visit or worse was maximized at a threshold of 3.2% predicted risk. At this threshold, the sensitivity of the need for urgent care visit or worse was 99.3% (95%CI, 98.3–99.7), specificity was 12.2% (95%CI, 11.2–13.2), PPV was 14.3% (95%CI, 13.3–15.4), and NPV was 99.2% (95%CI, 98.0–99.7). Using a threshold of 10% predicted risk for the same outcome, the sensitivity was 72.8% (95%CI, 69.1–76.1), specificity was 71.6% (95%CI, 70.2–72.9), PPV was 27.5% (95%CI, 25.3–29.7), and NPV was 94.7% (95%CI, 93.8–95.4). Finally, at a threshold of 10% predicted risk for hospitalization or worse, the sensitivity was 59.6% (95%CI 53.3–65.7), specificity was 90.8% (95%CI 89.9–91.6), PPV was 25.4% (95%CI 22.0–29.2) and NPV was 97.7% (95%CI 97.2–98.1). The performance of the model as a function of continuous predicted risk is shown in Supplementary Figure 10.

### Prognostic model usage

**Fig. 3:**
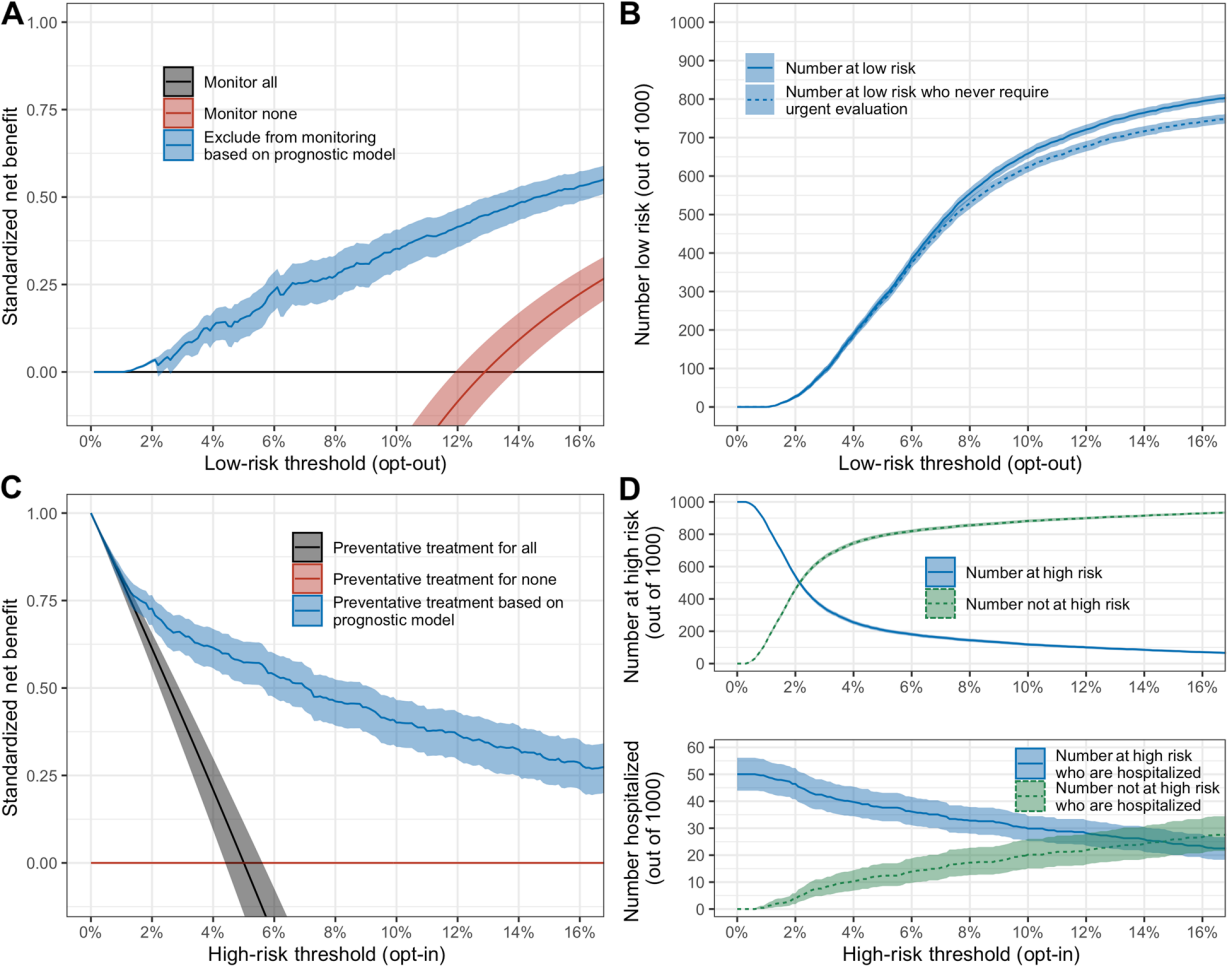
**A, C** Illustration of the standardized net benefit of the prognostic model (blue lines) compared with the strategies of monitoring or treating all individuals (black lines) and not providing any follow-up or treatment (red lines) over a range of risk thresholds. **B**, **D** The number of persons (out of 1000) who would be categorized at high or low risk for each risk threshold. **A** The use of the prognostic model to omit persons from follow-up who are at low risk of an urgent care visit or worse. The *Y*-axis represents the net increase in the proportion of low-risk individuals who avoid unnecessary monitoring (out of a hypothetical maximum achieved when the true negative rate is one and false negative rate is zero) compared with the strategy of enrolling all persons. **B** Illustration of the expected number of individuals (out of 1000) who would be omitted from monitoring using the prognostic model as a function of low-risk threshold (blue line) and the number of persons who would be omitted and who would never require an urgent in-person evaluation or worse (dashed blue line). **C** The use of the prognostic model to offer individuals who are at high risk of hospitalization or worse more rigorous follow-up or therapeutic intervention. The *Y*-axis represents the net increase in the proportion of high-risk individuals who are offered treatment (out of a hypothetical maximum achieved when the true positive rate is one and the false positive rate is zero) compared with the strategy of treating no cases. **D** The expected number of individuals (out of 1000) who would be offered treatment (blue line) and not be offered treatment (dashed green line) as a function of high-risk threshold. Also shown is the expected number of high-risk individuals (blue line) and those not at high risk (dashed green line) who later would have required hospitalization

The full model is presented in Supplementary Table 2. For the decision to omit individuals at low-risk for requiring an urgent in-person evaluation or worse from monitoring, a decision curve analysis revealed that the standardized net benefit of the prognostic model, compared with the strategy of enrolling all SARS-CoV-2-positive persons into monitoring, ranged from 0.07 to 0.31 over a reasonable span of low-risk thresholds (3% to 10%) (Fig. 3A). At these thresholds of low risk, one would expect to omit from monitoring between 93 and 658 out of every 1000 SARS-CoV-2-positive adults, of whom between 92 (98.9%) and 623 (94.6%) would never require urgent in-person evaluation (Fig. 3B). Similarly, for the decision to recommend comprehensive monitoring or early treatment for individuals at high risk of hospitalization or worse, the standardized net benefit of the model, compared with a strategy of not offering intensive management to anyone, ranged from 0.40 to 0.57 over a reasonable scope of high-risk thresholds (5 to 10%) (Fig. 3C). If individuals with a predicted risk of 5% or higher were considered high risk, one would expect to recommend intensive management for 208 (95%CI, 196–219) out of every 1000 infected adults, of whom 37 (95%CI, 33–43) would have later required hospitalization. At a threshold of 10% or higher, 118 (95%CI, 109–127) out of 1000 would be classified as high risk, of whom 30 (95%CI, 25–34) would be hospitalized (Fig. 3D). Decision curve analysis for the risk of ICU admission or death is shown in Supplementary Figure 11. The potential impact of incorporating the prognostic model in the clinical pathway of the telehealth service provided by the COVID-19 Outpatient Clinic is shown in Supplementary Figure 12.

## Discussion

In this nationwide study, a prognostic model was derived in a prospective population-based cohort of SARS-CoV-2-positive unvaccinated adults. We demonstrate how the model could be implemented in the context of a telehealth service, in which all the necessary information for prognostication is obtained through telephone interviews, followed by triage to the appropriate level of care.

The COVID-19 pandemic has in many cases overwhelmed national healthcare systems due to the large number of seriously ill patients requiring advanced medical care. High rates of virologic testing and isolation of positive individuals can assist in curtailing spread, but increases the number of identified cases. Although SARS-CoV-2 infection can lead to severe disease and death, most cases are mild or asymptomatic. Predicting who will subsequently require comprehensive care and, equally importantly, identifying those who will only need minimum or no further follow-up is challenging. However, potential solutions do exist, including optimization of healthcare resources allocation and reduction of the overall healthcare burden using early interventions. Risk stratifying patients in a pandemic setting with soaring case counts requires efficient and robust methods that should ideally be implemented without the need for in-person contact or extensive clinical testing. Limited evidence supporting such stratification is currently available.

Our prognostic model was derived in a large community-based cohort of adults that is considerably larger than most SARS-CoV-2-positive cohorts used for previously developed models, which have had a median of 338 cases [23]. On internal validation, our model was found to have good ability to discriminate between persons who either will or will not experience each of the three outcomes, as reflected by a high optimism-corrected C-statistic (Table 2). Although calibration metrics were excellent, visual examination of the calibration plots revealed a slight tendency to yield overly extreme predictions for individuals at high risk (Fig. 2). This was especially apparent for the outcome event ICU admission or death above a risk of approximately 20%, which likely resulted in part because only 30 individuals in our cohort had this predicted risk or higher. These extreme predictions would only have practical relevance if decision makers intended to use the prognostic model at this or higher threshold for this particular outcome event. However, in Supplementary Figure 11 we show that the standardized net benefit of the prognostic model at high-risk thresholds at or above 16% is zero to negative compared with a strategy of treating no patient irrespective of risk. Using the prognostic model at such thresholds would therefore seem to be inadvisable regardless of calibration. Even so, it should be stated that if future predictions appear at or above this level of risk they should be considered to be of unknown accuracy.

In addition to demonstrating good discrimination and calibration of the prognostic model, we report the clinical utility of the model based on decision curve analysis. The optimal thresholds at which the prognostic model should be used for resource allocation will vary based on local factors, such as the number of persons that are diagnosed in relation to the capacity of the healthcare infrastructure and the costs and benefits of the treatments or monitoring strategies being considered. The rationale for basing the decision of whom to monitor on the risk of urgent care visit or worse, is the need to minimize unexpected clinic visits of SARS-CoV-2-positive persons to avoid potential transmission to other patients and healthcare workers. By monitoring those at risk, urgent visits can be managed more effectively. We show that the prognostic model can identify low-risk individuals who could be safely omitted from telehealth monitoring. Conversely, early therapeutic interventions such as administration of monoclonal antibodies against SARS-CoV-2 [24] or antiviral drugs, should be prioritized for those who are at high risk of subsequent hospitalization. We again show that the prognostic model can identify a large proportion of those who will later require hospitalization for COVID-19.

A strong practical advantage of our prognostic model is the ability to estimate the risk of several important clinical outcomes using predictor variables that can be collected by telephone interview at the time of diagnosis, as opposed to conventional clinical assessment that usually includes physical examination, laboratory testing and imaging studies. The model was derived in the setting of easy access to diagnostic testing for SARS-CoV-2, and included all adults in Iceland who were not hospitalized at the time of diagnosis of SARS-CoV-2 infection and in whom language barriers would not prevent effective telephone interview. The risk of selection bias is therefore minimal. Furthermore, data were invariably obtained by trained healthcare providers within 24 h of the individual’s first positive qPCR test using a standardized questionnaire, thereby minimizing recall bias. A systematic review by Wynants et al. compared 107 models that predicted progression to severe disease or mortality from COVID-19 [7]. The authors identified only two prognostic models that were associated with low risk of bias, the 4C mortality score and QCOVID. The 4C mortality score was developed in a cohort of hospitalized patients with COVID-19 and is intended to be used at the time of hospitalization [25]. It included predictor variables that would require in-person evaluation by trained healthcare workers (respiratory rate, peripheral oxygenation and Glascow coma scale) and laboratory findings (urea and CRP) that would necessitate a blood draw. This model therefore would not be helpful in performing triage of SARS-CoV-2-positive adults in the community at the time of diagnosis and could not inform the decision to omit individuals at low risk of requiring urgent care visits from telehealth monitoring, nor the decision to provide early treatment to individuals at high risk of hospitalization. The QCOVID model was developed based on data collected from population-based registries and is meant to predict the time until death and hospitalization at the population level, rather than solely for persons with a confirmed SARS-CoV-2 infection [8, 9]. It does not predict the need for urgent outpatient evaluation, and some of the included predictors, such as the Townsend deprivation score, are difficult to implement in populations outside of the UK. Among the remaining 105 prognostic models at high or unknown risk of bias, common causes of bias were small and non-representative study cohorts in which inclusion was unclear, and many individuals were excluded because they had not developed an outcome by the end of the study period, i.e., they had not recovered, been discharged from hospital, or died [7]. In the current study, follow-up of all cases was complete, as they were only discharged from monitoring if at least 14 days had passed from qPCR-based diagnosis and at least 7 days had passed from resolution of symptoms, resulting in a median duration of follow-up time of 15 days and up to 64 days for patients with persistent symptoms. Finally, the reporting of our prognostic model derivation adhered to the TRIPOD guidelines. For these reasons, we believe our model adds to the existing literature as a potential tool for clinical decision-making.

While the strength of the present study resides in the population-based approach, there are several noteworthy limitations. The clinical threshold at which an urgent care visit is considered to be indicated will likely vary between cultures and healthcare systems. This variation in clinical thresholds also exists for hospitalizations and ICU admissions, but is probably considerably smaller than for outpatient evaluation. In addition, the clinical severity score was loosely defined and was largely based on the clinical impression of the physician or nurse conducting the enrollment interview. Inter-rater variability was not quantified and we are therefore unable to speculate how this predictor will generalize to other settings. As the need for urgent in-person evaluation at the COVID-19 Outpatient Clinic was also determined by the same healthcare professionals during telephone interviews, it is perhaps not surprising that the clinical severity score was predictive. However, the clinical severity score during the enrollment interview also predicted the need for hospital admission, ICU care and death. Furthermore, a study examining the humoral response to SARS-CoV-2 in an overlapping cohort found a strong correlation between the clinical severity score and higher anti-SARS-CoV-2 antibody levels, supporting the validity of the clinical severity score [6]. Finally, our prognostic model was developed in 2020, before vaccination for SARS-CoV-2 became available. The predicted risks are therefore only accurate for unvaccinated adults and should be considered an upper-limit estimate for those who have received vaccination. Further studies are needed to evaluate the prognostic model in vaccinated persons.

## Conclusion

Our multivariable prognostic model predicts the risk of COVID-19-related urgent care visit, hospitalization, and ICU admission or death, for an unselected group of unvaccinated SARS-CoV-2-positive adults based on symptoms at diagnosis, comorbidities and demographic factors. These variables can be sampled by telephone interview at the time of diagnosis of SARS-CoV-2 infection. This information may be valuable for risk stratification of cases at the time of diagnosis and prioritization of health care resources.

## Supplementary Information

**Additional file 1.**

## Data Availability

The data that support the findings of this study are available from the corresponding authors on reasonable request.

## References

[1] Zhu N, Zhang D, Wang W, Li X, Yang B, Song J (2020). A novel coronavirus from patients with pneumonia in China, 2019. N Engl J Med..

[2] Huang C, Wang Y, Li X, Ren L, Zhao J, Hu Y (2020). Clinical features of patients infected with 2019 novel coronavirus in Wuhan, China. Lancet..

[3] Verity R, Okell LC, Dorigatti I, Winskill P, Whittaker C, Imai N (2020). Estimates of the severity of coronavirus disease 2019: a model-based analysis. Lancet Infect Dis..

[4] Blackburn J, Yiannoutsos CT, Carroll AE, Halverson PK, Menachemi N (2021). Infection fatality ratios for COVID-19 among noninstitutionalized persons 12 and older: results of a random-sample prevalence study. Ann Intern Med..

[5] Helgason D, Eythorsson E, Olafsdottir LB, Agustsson T, Ingvarsdottir S, Sverrisdottir S (2021). Beating the odds with systematic individualized care: Nationwide prospective follow-up of all patients with COVID-19 in Iceland. J Intern Med..

[6] Gudbjartsson DF, Norddahl GL, Melsted P, Gunnarsdottir K, Holm H, Eythorsson E (2020). Humoral immune response to SARS-CoV-2 in Iceland. N Engl J Med..

[7] Wynants L, Calster BV, Collins GS, Riley RD, Heinze G, Schuit E (2020). Prediction models for diagnosis and prognosis of covid-19: systematic review and critical appraisal. BMJ.

[8] Clift AK, Coupland CAC, Keogh RH, Diaz-Ordaz K, Williamson E, Harrison EM (2020). Living risk prediction algorithm (QCOVID) for risk of hospital admission and mortality from coronavirus 19 in adults: national derivation and validation cohort study. BMJ..

[9] Nafilyan V, Humberstone B, Mehta N, Diamond I, Coupland C, Lorenzi L (2021). An external validation of the QCovid risk prediction algorithm for risk of mortality from COVID-19 in adults: a national validation cohort study in England. Lancet Digit Health..

[10] Hippisley-Cox J, Coupland CA, Mehta N, Keogh RH, Diaz-Ordaz K, Khunti K (2021). Risk prediction of covid-19 related death and hospital admission in adults after covid-19 vaccination: national prospective cohort study. BMJ..

[11] Levey AS, Stevens LA, Schmid CH, Lucy ZY, Castro AF, Feldman HI (2009). A new equation to estimate glomerular filtration rate. Ann Intern Med..

[12] Riley RD, Snell KI, Ensor J, Burke DL, Harrell FE, Moons KG (2019). Minimum sample size for developing a multivariable prediction model: PART II - binary and time-to-event outcomes. Stat Med..

[13] Riley RD, Ensor J, Snell KIE, Harrell FE, Martin GP, Reitsma JB (2020). Calculating the sample size required for developing a clinical prediction model. BMJ..

[14] Harrell FE Jr. Regression modeling strategies. 2nd ed. Cham: Springer International Publishing; 2016.

[15] Harrell FE, Margolis PA, Gove S, Mason KE, Mulholland EK, Lehmann D (1998). Development of a clinical prediction model for an ordinal outcome: The World Health Organization Multicentre Study of Clinical Signs and Etiological Agents of Pneumonia, Sepsis and Meningitis in Young Infants. Stat Med..

[16] Van Calster B, Nieboer D, Vergouwe Y, De Cock B, Pencina MJ, Steyerberg EW (2016). A calibration hierarchy for risk models was defined: from utopia to empirical data. J Clin Epidemiol..

[17] Bartlett JW, Hughes RA (2020). Bootstrap inference for multiple imputation under uncongeniality and misspecification. Stat Methods Med Res..

[18] Vickers AJ, Elkin EB (2006). Decision curve analysis: A novel method for evaluating prediction models. Med Decis Mak.

[19] Moons KG, Altman DG, Reitsma JB, Ioannidis JP, Macaskill P, Steyerberg EW (2015). Transparent Reporting of a multivariable prediction model for Individual Prognosis Or Diagnosis (TRIPOD): explanation and elaboration. Ann Intern Med.

[20] R Core Team (2020). R: A language and environment for statistical computing [Internet].

[21] Wickham H, Averick M, Bryan J, Chang W, McGowan LD, François R (2019). Welcome to the tidyverse. J Open Source Softw..

[22] Wilke CO. Cowplot: Streamlined plot theme and plot annotations for ‘ggplot2’ [Internet]. 2019. Available from: https://CRAN.R-project.org/package=cowplot

[23] Yoshida K, Bartel A. Tableone: Create ‘Table 1’ to describe baseline characteristics with or without propensity score weights [Internet]. 2020. Available from: https://CRAN.R-project.org/package=tableone

[24] Weinreich DM, Sivapalasingam S, Norton T, Ali S, Gao H, Bhore R (2021). REGEN-COV antibody combination and outcomes in outpatients with Covid-19. N Engl J Med..

[25] Knight SR, Ho A, Pius R, Buchan I, Carson G, Drake TM (2020). Risk stratification of patients admitted to hospital with covid-19 using the ISARIC WHO Clinical Characterisation Protocol: Development and validation of the 4C Mortality Score. BMJ..

