# Supplementary material for: Development of a prognostic model of COVID-19 severity: a population-based cohort study in Iceland
Source: Diagn Progn Res. 2022 Sep 8;6:17. doi: 10.1186/s41512-022-00130-0 (PMC9451645; doi:10.1186/s41512-022-00130-0)
Supplement: Supplementary file 1 — Additional file 1. [file 41512_2022_130_MOESM1_ESM.docx]

**Supplementary Methods**

***Model discrimination and calibration indices***

Model discrimination was quantified using the C-statistic (the probability that a randomly selected individual who did experience the outcome has a higher predicted probability than a randomly selected individual who did not). Calibration was assessed by visual examination of calibration plots and by using several calibration indices: outcome specific calibration intercept (assessment of mean risk with a target value of 0, such that negative values represent systematic overestimation of risk and positive values systematic underestimation of risk), overall calibration slope (assessment of estimated risk with a target value of 1, such that values <1 represent estimated risks that are too extreme [too high for high-risk persons and too low for low-risk persons] and values >1 represent estimated risks that are too conservative [too low for high-risk persons and too high for low-risk persons]), outcome specific Brier score (the mean squared difference between the predicted probability of the outcome for each person and his/her observed outcome, which ranges from 0 to 1, such that 0 indicates perfect accuracy and 1 perfect inaccuracy), and E_max_ (the maximum absolute difference between the predicted probability of the outcome and the LOWESS calibrated probability over the range of predicted probability from 0 to 1).

***Decision curve analysis***

To aid in determining whether an individual’s risk of clinical deterioration requiring an urgent care visit or worse is sufficiently small to omit from telehealth follow-up, the standardized net benefit of the prognostic model at a low-risk threshold L is defined as ${TNR}_{L}-\frac{p}{1-p}*\frac{1-L}{L}*{FNR}_{L}$ where *TNR_L_* is the true negative rate (the rate of individuals classified as low-risk who did not require an urgent care visit or worse)*, p* is the proportion of individuals who required an urgent care visit or worse, and *FNR_L_* is the false negative rate (the rate of individuals classified as low risk who did require an urgent care visit or worse). This can alternatively be stated as the net increase in the proportion of low-risk individuals who avoided unnecessary follow-up using the prognostic model compared with a strategy of enrolling all individuals into the telehealth service, standardized to the maximum theoretically possible value of net benefit, which would occur when the $TNR=1$ and $FNR=0$. To aid in determining whether an individual’s risk of hospital admission or worse is sufficiently large to justify providing more intensive treatment, the standardized net benefit was defined as ${TPR}_{H}-\frac{1-p}{p}*\frac{H}{1-H}*{FPR}_{H}$for a high-risk threshold *H,* where *TPR* is the true positive rate (the rate of individuals classified as high risk among those who were hospitalized or died), *p* is the proportion of individuals who were hospitalized or died and *FPR* is the false positive rate (the rate of individuals classified as high risk among those who did not require hospitalization and did not die), which can alternatively be stated as the net increase in the proportion of individuals who would be appropriately be provided with early treatment compared with a strategy of not offering early treatment to any individual, standardized to the maximum theoretically possible value of net benefit, which would occur when the $TPR=1$ and $FPR=0$.

**Supplementary Tables**

**Supplementary Table 1.** The definition of each variable used in the prognostic model, multiple imputation procedure or risk factor analysis.

| **Predictor** | **Variable description** | **Variable use** |
| --- | --- | --- |
| Age | Age of the person in years at the time of the first positive qPCR test. In the prognostic and risk factor analysis, age was modelled with a restricted cubic spline with knots placed at the 0.05, 0.35, 0.65 and 0.95 percentiles. For the multiple imputation procedure, a restricted cubic spline with six knots at the 0.05, 0.23, 0.41, 0.59, 0.77 and 0.95 percentiles was used. | Prognostic model, multiple imputation for prognostic model |
| Sex | Sex of the person as recorded in the Icelandic population register. Female = 0, Male = 1. | Prognostic model, multiple imputation for prognostic model |
| Body mass index (BMI) | Weight (kg) divided by height squared (cm) as reported during the enrollment interview. The prognostic model included BMI as a linear predictor. The multiple imputation procedure included BMI using a restricted cubic spline with six knots placed at the 0.05, 0.23, 0.41, 0.59, 0.77 and 0.95 percentiles. | Prognostic model, multiple imputation for prognostic model |
| Hypertension,  prospective | History of hypertension reported during the enrollment interview. Absent = 0,  Present = 1. | Prognostic model, multiple imputation for prognostic model |
| Diabetes,  prospective | History of diabetes reported during the enrollment interview. Absent = 0,  Present = 1. | Prognostic model, multiple imputation for prognostic model |
| Heart disease | History of any heart disease reported during the enrollment interview. Absent = 0,  Present = 1. | Prognostic model, Multiple imputation for prognostic model |
| Pulmonary disease,  prospective | History of any pulmonary disease reported during the enrollment interview. Absent = 0,  Present = 1. | Prognostic model, multiple imputation for prognostic model |
| Current or prior malignancy,  prospective | History of current or prior malignancy reported during the enrollment interview. Absent = 0,  Present = 1. | Prognostic model, multiple imputation for prognostic model |
| Current smoking | Current cigarette smoking reported during the enrollment interview. Absent = 0,  Present = 1. | Prognostic model, multiple imputation for prognostic model |
| Flu-like symptoms | Presence of any one of the following symptoms reported during the enrollment interview: fever (≥38°C), chills or rigors, non-productive cough, headache, lethargy, myalgia or anorexia. Absent = 0,  Present = 1.v | Prognostic model, multiple imputation for prognostic model |
| Upper respiratory symptoms | Presence of any one of the following symptoms reported during the enrollment interview: rhinorrhea, sore throat, dysosmia or dysgeusia. Absent = 0,  Present = 1. | Prognostic model, multiple imputation for prognostic model |
| Lower respiratory symptoms | Presence of any one of the following symptoms reported during the enrollment interview: productive cough, shortness of breath or dyspnea on exertion or at rest. Absent = 0,  Present = 1. | Prognostic model, multiple imputation for prognostic model |
| Gastrointestinal symptoms | Presence of any one of the following symptoms reported during the enrollment interview: nausea, vomiting, abdominal pain or diarrhea. Absent = 0,  Present = 1. | Prognostic model, multiple imputation for prognostic model |
| Clinical severity score | The clinical severity of the current illness as judged by the nurse or physician conducting the enrollment interview, loosely defined as: low severity (mild symptoms), moderate severity (mild dyspnea, cough or fever less than five days) and high severity (severe dyspnea, worsening cough and high or persistent fever for five days or longer).  Mild = 0, moderate or high = 1. | Prognostic model, multiple imputation for prognostic model |
| Hypertension, registry-based | ICD-10 diagnosis code I10-I16 specifying hypertensive disease or subgroups recorded in Landspitali–The National University Hospital of Iceland‘s patient registry in 2009-2020, the Register of Primary Health Care Contacts in 2004-2020 or the Register of Contacts with Medical Specialists in Private Practice in 2010-2020 **AND** a filled prescription for a medication assigned ATC code C02 (antihypertensive drugs), C07 (beta blocking agents), C08 (calcium channel blockers), or C09 (drugs acting on the renin-angiotensin system), recorded in the Prescription Medicines Register  between 395 days and 14 days before the the person´s first positive qPCR test. Absent = 0,  Present = 1. | Multiple imputation for prognostic model |
| Diabetes, registry-based | ICD-10 diagnosis code E08-13 specifying diabetes mellitus or subgroups recorded in Landspitali–The National University Hospital of Iceland‘s patient registry in 2009-2020, the Register of Primary Health Care Contacts in 2004-2020 or the Register of Contacts with Medical Specialists in Private Practice in 2010-2020 **AND** a filled prescription for a medication assigned ATC code A10 and subgroups (antidiabetic drugs) recorded in the Prescription Medicines Register between 395 days and 14 days before the person´s first positive qPCR test. Absent = 0,  Present = 1. | Multiple imputation for prognostic model |
| Heart disease, registry-based | ICD-10 diagnosis codeI20-I25 specifying ischemic heart disease, I50 specifying heart Failure, I34-I37 specifying nonrheumatic valve diseases, or I44-I49 specifying arrhythmias or subgroups recorded in Landspitali–The National University Hospital of Iceland‘s patient registry in 2009-2020, the Register of Primary Health Care Contacts in 2004-2020 or the Register of Contacts with Medical Specialists in Private Practice in 2010-2020 **AND** a filled prescription for a medication assigned ATC code B01AC (platelet aggregation inhibitors excl. heparin), C10 (lipid modifying agents), C01DA (organic nitrates), C03 (diuretics), C07 (beta blocking agents) or C09 (drugs acting on the renin-angiotensin system) recorded in the Prescription Medicines Register between 395 days and 14 days before the person´s first positive qPCR test. Absent = 0,  Present = 1. | Multiple imputation for prognostic model |
| Chronic kidney disease, registry-based | Chronic kidney disease defined as the presence of two separate eGFR determinations <60 mL/min/1.73 m^2^ that were obtained at least 90 days apart, and did not occur during an episode of acute kidney injury.  The eGFR was calculated from serum creatinine values obtained from the Landspitali–The National University Hospital of Iceland‘s patient registry in 2009-2020 and the Register of Primary Health Care Contacts in 2004-2020, using the Chronic Kidney Disease Epidemiology Collaboration equation. Absent = 0,  Present = 1. | Multiple imputation for prognostic model |
| Pulmonary disease, registry-based | ICD-10 diagnosis code J40-J47 specifying chronic lower respiratory diseases or subgroups, J60-J70 specifying lung diseases due to external agents or subgroups, or J80-J84 specifying other respiratory diseases principally affecting the interstitium or subgroups recorded in Landspitali–The National University Hospital of Iceland‘s patient registry in 2009-2020, the Register of Primary Health Care Contacts in 2004-2020 or the Register of Contacts with Medical Specialists in Private Practice in 2010-2020 **AND** a filled prescription for medication assigned ATC code R03 (drugs for obstructive airway diseases) recorded in the Prescription Medicines Register between 395 days and 14 days before the person´s first positive qPCR test. Absent = 0,  Present = 1. | Multiple imputation for prognostic model |
| Current or prior malignancy, registry-based | ICD-10 diagnosis code C00-C96 specifying neoplasms or subgroups recorded in Landspitali–The National University Hospital of Iceland‘s patient registry in 2009-2020, the Register of Primary Health Care Contacts in 2004-2020 or the Register of Contacts with Medical Specialists in Private Practice in 2010-2020. Absent = 0,  Present = 1. | Multiple imputation for prognostic model |
| Time from first case | Elapsed time in days from the day before the first diagnosed case of SARS-CoV-2 infection in Iceland (February 26, 2020) to the date of the person´s first positive qPCR test. This was modelled with a restricted cubic spline with six knots at the 0.05, 0.23, 0.41, 0.59, 0.77 and 0.95 percentiles. | Multiple imputation for prognostic model |
| Time from symptoms to diagnosis | Elapsed time in days from date of symptom onset as reported during the enrollment interview to the date of the person´s first positive qPCR test. This was modelled with a restricted cubic spline with six knots at the 0.05, 0.23, 0.41, 0.59, 0.77 and 0.95 percentiles. | Multiple imputation for prognostic model |
| Follow-up time | Elapsed time in days from the telehealth service enrollment interview to termination of telehealth follow-up or death. This was modelled with a restricted cubic spline with six knots at the 0.05, 0.23, 0.41, 0.59, 0.77 and 0.95 percentiles. | Multiple imputation for prognostic model |
| Number of primary care visits during the preceding two years | The number of visits to primary care services recorded in the Register of Primary Health Care Contacts between 730 days and 14 days before the person’s first positive qPCR test. This was modelled with a restricted cubic spline with six knots at the 0.05, 0.23, 0.41, 0.59, 0.77 and 0.95 percentiles. | Multiple imputation for prognostic model |
| Prior hospitalization | Whether or not the patient had been admitted to Landspitali–The National University Hospital of Iceland between 730 days and 14 days before the first positive qPCR test. Absent = 0,  Present = 1. | Multiple imputation for prognostic model |
| Number of filled prescriptions during the preceding 395 days (13 months) | The number of filled prescriptions n the Prescription Medicines Register between 395 days and14 days before a patients’ first positive qPCR test. This was modelled with a restricted cubic spline with six knots at the 0.05, 0.23, 0.41, 0.59, 0.77 and 0.95 percentiles. | Multiple imputation for prognostic model |
| Residence in Capital Region | The residence of the patient at the time of his/her first positive qPCR test. Equal to one if the patient resides within the Capital Region, and zero otherwise. | Multiple imputation for prognostic model |

**Supplementary Table 2.** The full prognostic model. The corresponding linear predictor function is the following: {+0.076618283 * age - 0.00011067312 * pmax(age - 20, 0) ^ 3 + 0.00025910235 * pmax(age - 32, 0) ^ 3 - 0.00019547598 * pmax(age - 48, 0) ^ 3 + 4.7046751e-05 * pmax(age - 70.25, 0) ^ 3 - 0.21253471 * sex + 0.041083947 * bmi_prospective - 0.073387976 * htn_prospective - 0.0073864975 * heart_prospective + 0.51728673 * pulm_prospective + 0.42773753 * diabetes_prospective - 0.030070485 * cancer_prospective + 0.023551294 * smoking_current + 0.53460995 * flulike_symptoms - 0.13557069 * upper_respiratory + 0.3023864 * lower_respiratory + 0.32132686 * gastrointestinal + 1.5898032 * clinical_score_fct}.

| Variable | Coefficient | Standard error |
| --- | --- | --- |
| y>=1 | -6.4137 | 0.7005 |
| y>=2 | -7.6866 | 0.7055 |
| y>=3 | -9.5440 | 0.7223 |
| age | 0.0766 | 0.0238 |
| age’ | -0.2795 | 0.0928 |
| age’’ | 0.6542 | 0.1878 |
| sex | -0.2125 | 0.0980 |
| bmi_prospective | 0.0411 | 0.0113 |
| htn_prospective | -0.0734 | 0.1368 |
| heart_prospective | -0.0074 | 0.1704 |
| pulm_prospective | 0.5173 | 0.1609 |
| diabetes_prospective | 0.4277 | 0.2222 |
| cancer_prospective | -0.0301 | 0.2379 |
| smoking_current | 0.0236 | 0.1833 |
| flulike_symptoms | 0.5346 | 0.1645 |
| upper_respiratory | -0.1356 | 0.1056 |
| lower_respiratory | 0.3024 | 0.1109 |
| gastrointestinal | 0.3213 | 0.1118 |
| clinical_score_fct | 1.5898 | 0.1244 |

**Supplementary Table 3.** Calibration and discrimination indices of the prognostic model for the derivation cohort, shown for each of the outcomes. Also shown are the optimism and optimism-corrected indices. The 95% bootstrapped confidence intervals are presented within parentheses.

| Indexes | Outcome | Value | Optimism | Optimism-corrected value |
| --- | --- | --- | --- | --- |
| C-statistic | - | 0.798 (0.795-0.802) | 0.0107 (0.00753-0.0141) | 0.793 (0.789-0.797) |
| Negalkerke’s R^2^ | - | 0.242 (0.236-0.250) | 0.00816 (0.00564-0.0106) | 0.234 (0.227- 0.242) |
| Calibration intercept | Urgent care | 0 | 0.0427 (0.0225-0.0636) | -0.043 (-0.064- -0.023) |
|  | Hospitalization | 0 | 0.0626 (0.0327 – 0.0941 | -0.063 (-0.094 –  -0.033) |
|  | ICU or death | 0 | 0.0981 (0.0417 – 0.155) | -0.098 (-0.155 –  -0.042) |
| Calibration slope | - | 1 | 0.0269 (0.0171-0.0366) | 0.973 (0.963-0.983) |
| Brier score | Urgent care | 0.0912 (0.0905 – 0.0919) | -0.00100 (-0.00151 –  -0.0000478 | 0.092 (0.091 - 0.093) |
|  | Hospitalization | 0.0384 (0.0379 – 0.0389) | -0.000428 (-0.000799 –  -0.00000207) | 0.039 (0.038 - 0.039) |
|  | ICU or death | 0.00997 (0.00976 – 0.0102) | -0.0000902 (-0.000308 – 0.000120) | 0.010 (0.010 - 0.010) |
| E_max_ | Urgent care | 0 | 0.0140 (0.00808 – 0.201) | 0.014 (0.008 - 0.020) |
|  | Hospitalization | 0 | 0.0183 (0.0103 – 0.0269) | 0.018 (0.010 - 0.027) |
|  | ICU or death | 0 | 0.0267 (0.0125 – 0.0412) | 0.027 (0.013 - 0.041) |

**Supplementary Figures**

**
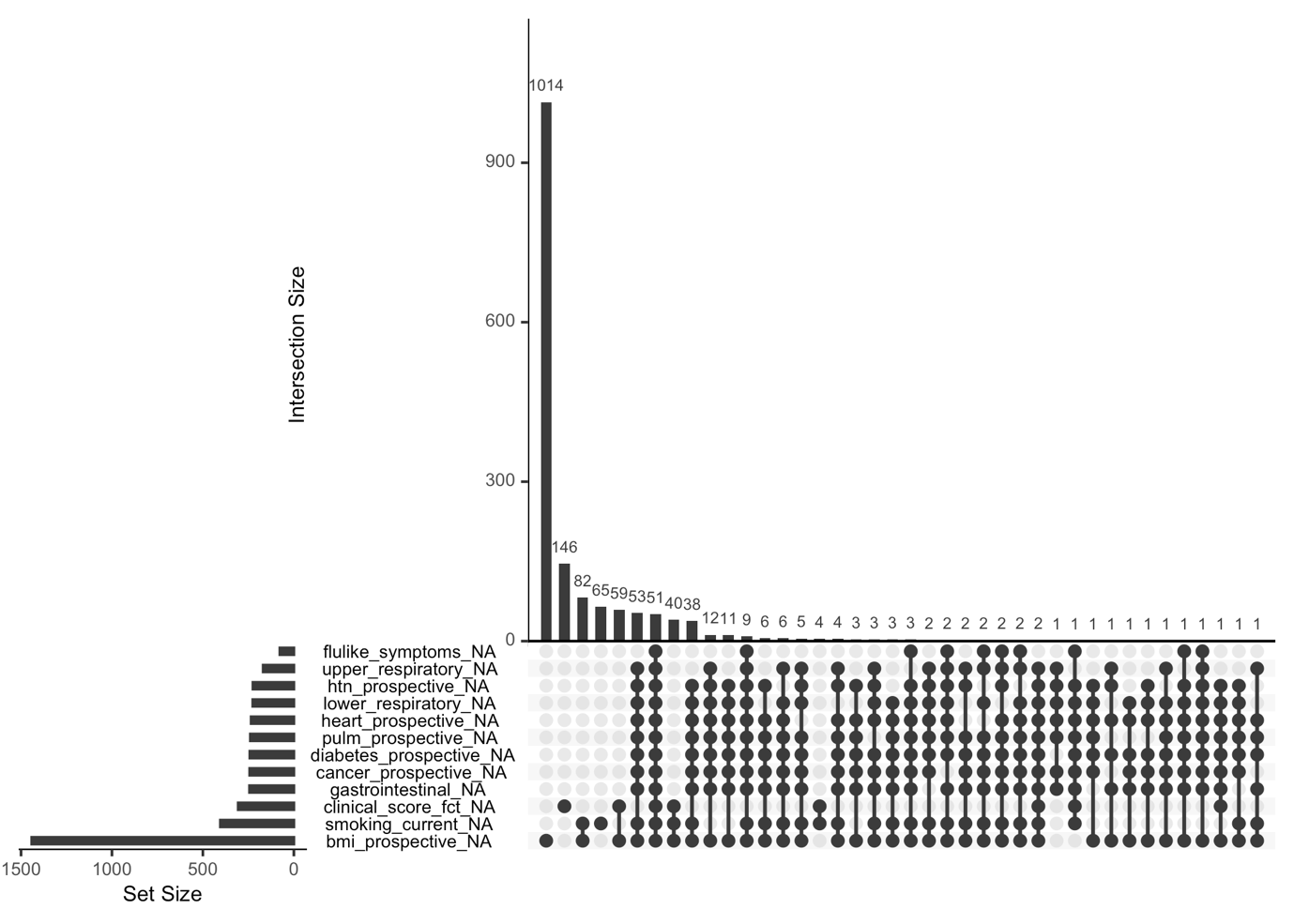
**

**Supplementary Figure 1.** The number of missing values of each predictor and predictor combination in the derivation cohort.

.

**
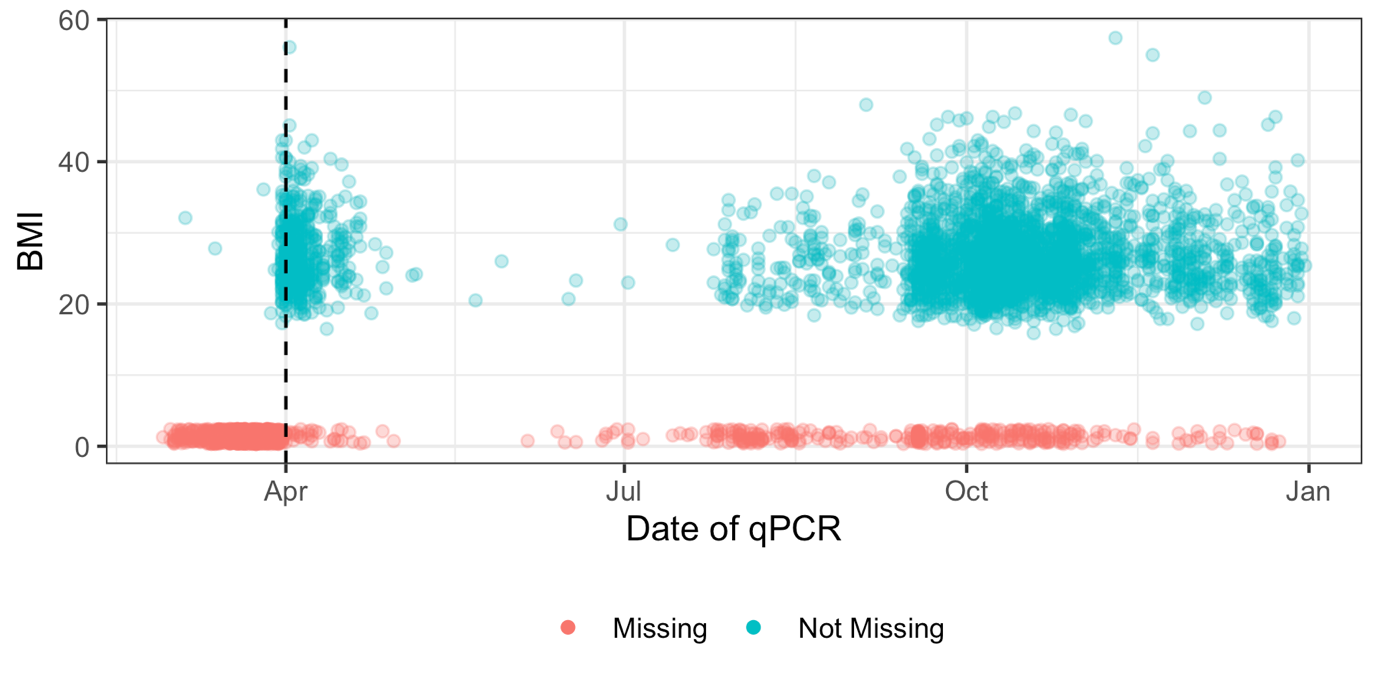
**

**Supplementary Figure 2.** The distribution of missing and non-missing body mass index (BMI) values as a function of the date of polymerase chain reaction (qPCR) result. The vertical dashed line on April 1 2020 illustrates the date at which BMI was added to the standardized questionnaire used during the enrollment interview of the telehealth service of the COVID-19 Outpatient Clinic of LUH. In total, 75% of missing BMI values occurred in patients who were qPCR positive before BMI was added to the questionnaire and can therefore be considered missing at random given qPCR date.


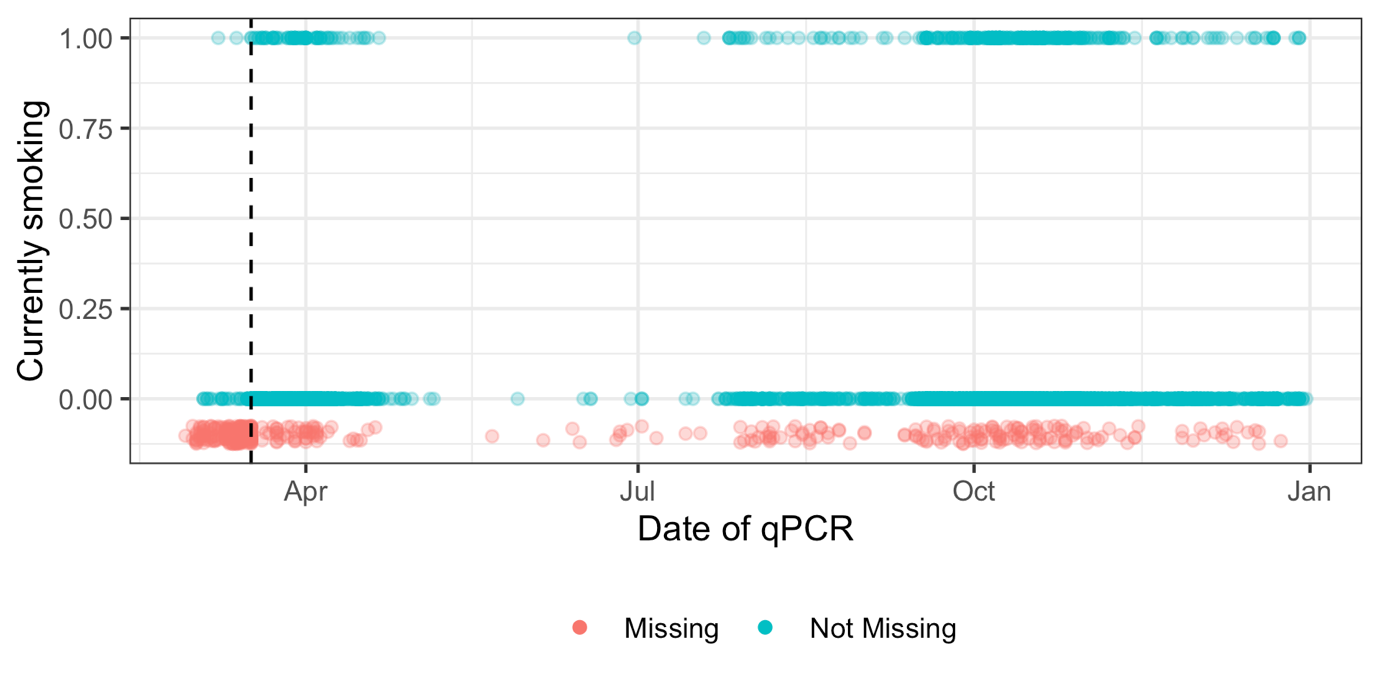


**Supplementary Figure 3.** The distribution of missing and non-missing values of smoking status as a function of the date of polymerase chain reaction (qPCR) result. The vertical dashed line on March 17 2020 illustrates the date at which a standardized questionnaire was implemented and used during the enrollment interview of the telehealth service of the COVID-19 Outpatient Clinic of LUH. In total, 50% of missing values occurred in patients who were qPCR positive before the questionnaire was implemented and can therefore be considered missing at random given qPCR date.


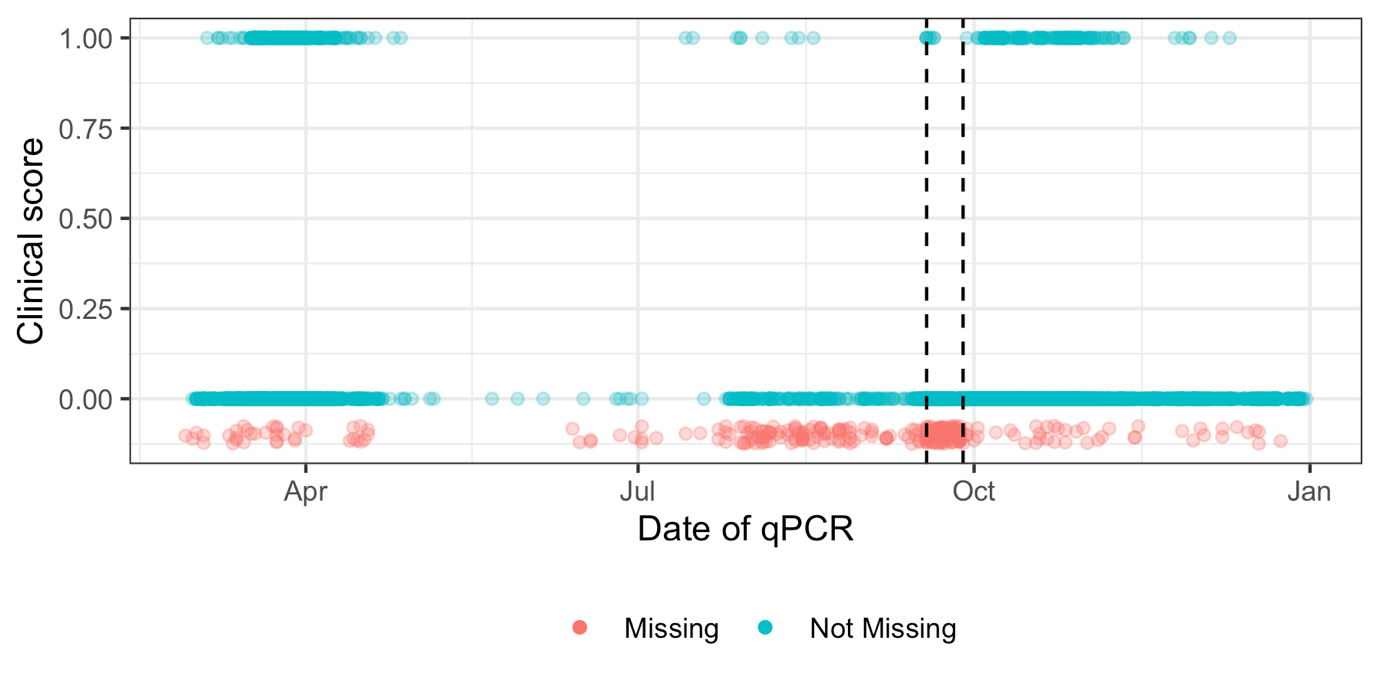


**Supplementary Figure 4.** The distribution of missing and non-missing values of smoking status as a function of the date of polymerase chain reaction (qPCR) result. The vertical dashed lines on September 18 and September 27 2021 illustrate the period during which a new staff were temporarily recruited to conduct enrollment interviews and neglected to record clinical score. In total, 30% of missing values occurred in patients who were qPCR during this period and can therefore be considered missing at random given qPCR date.

**
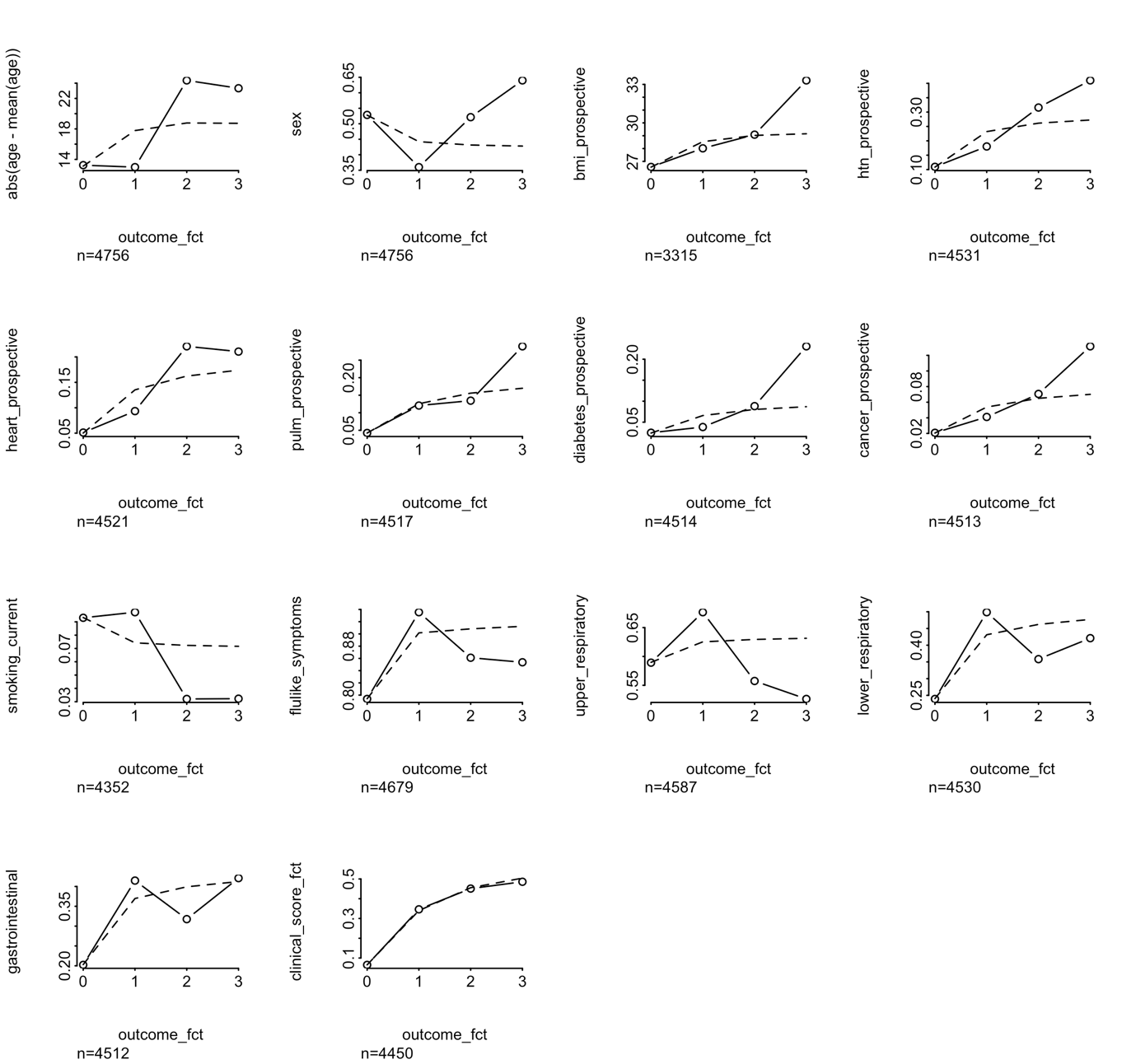
**

**Supplementary Figure 5.** The unadjusted mean values of each predictor at the different outcome levels (0 = absence of clinical deterioration requiring in-person evaluation or hospitalization for the duration of telehealth care; 1 = clinical deterioration requiring urgent in-person evaluation at the LUH COVID-19 Outpatient Clinic, but not subsequent hospitalization; 3 = hospitalization; and 4 = admission to intensive care unit (ICU) or death). Because age is highly non-linear, it is represented as the mean absolute difference of age and its mean. The solid line connects the observed mean values, and the dashed line depicts what the expected mean values would be if the proportional odds assumption were satisfied.


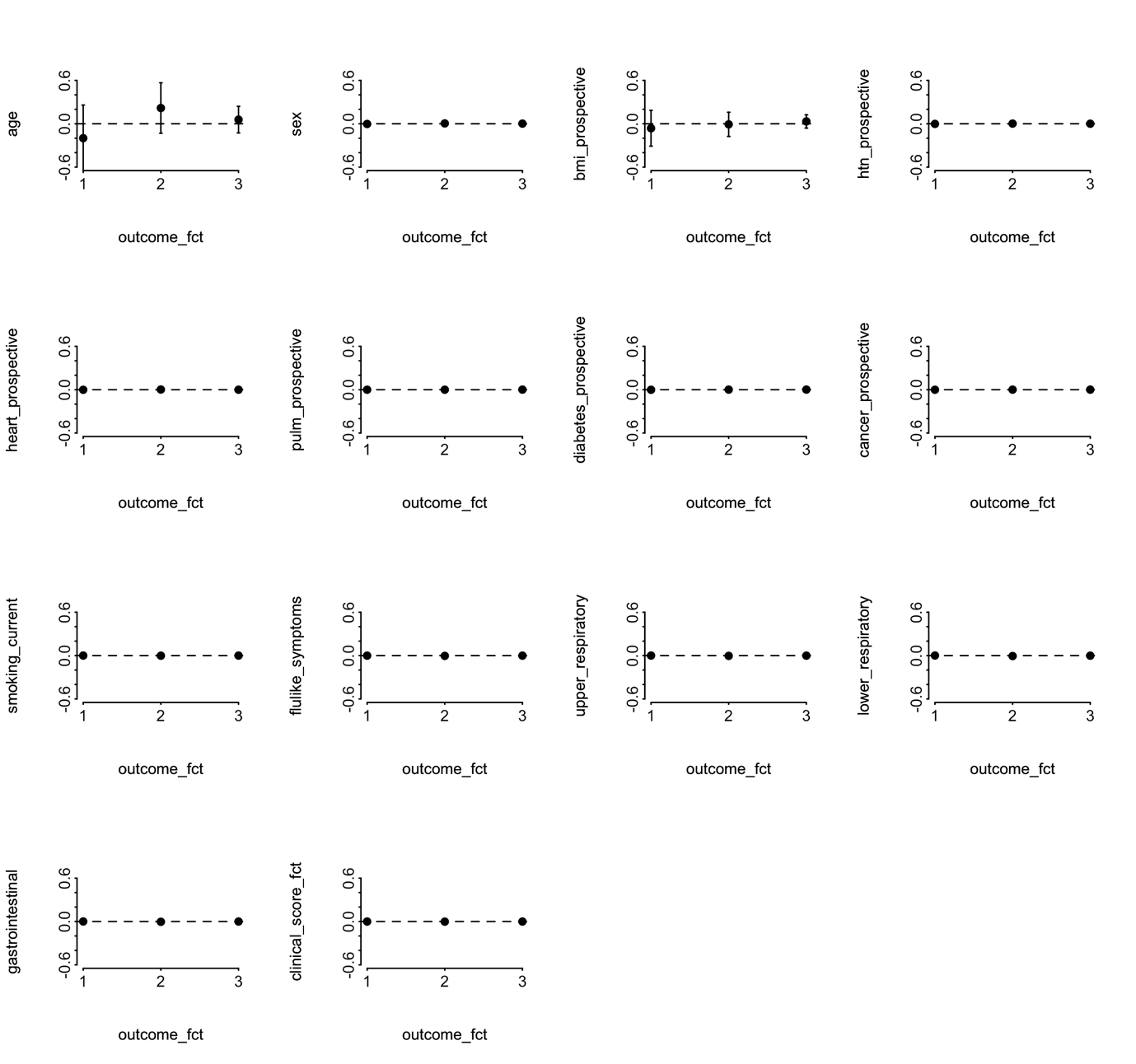


**Supplementary Figure 6.** Binary score residuals for each of the outcomes (1 = clinical deterioration requiring urgent in-person evaluation at the LUH COVID-19 Outpatient Clinic, but not subsequent hospitalization; 2 = hospitalization; and 3 = admission to intensive care unit (ICU) or death). to assess the proportional odds assumption. Here age is modeled linearly. There is moderate evidence of violation of the proportional odds assumption for the predictors *sex, smoking_current, lower_respiratory,* and *gastrointestinal.* However, the binary score residuals for these predictors are two orders of magnitude lower than for *age* and *bmi_prospective*, which are both consistent with the proportional odds assumption.


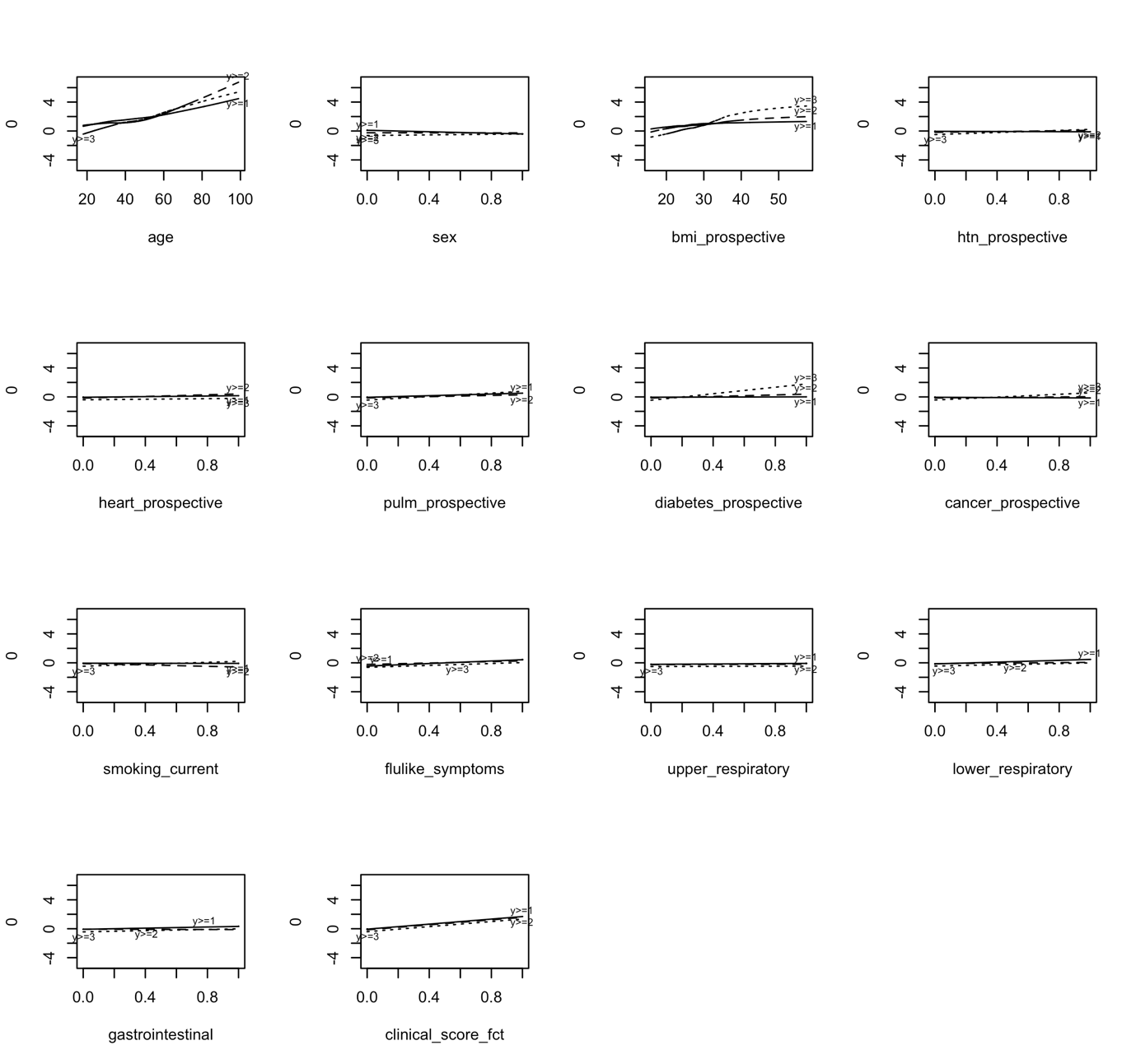


**Supplementary Figure 7.** Smoothed partial residuals (Y-axis) over predictor values (X-axis). Three lines are shown, one for each outcome (1 = clinical deterioration requiring urgent in-person evaluation at the LUH COVID-19 Outpatient Clinic, but not subsequent hospitalization; 3 = hospitalization; and 4 = admission to intensive care unit (ICU) or death). to assess the proportional odds assumption. Here age is modeled linearly. There is moderate evidence of violation of the proportional odds assumption for the predictors *sex, flulike_symptoms, lower_respiratory,* and *gastrointestinal.*


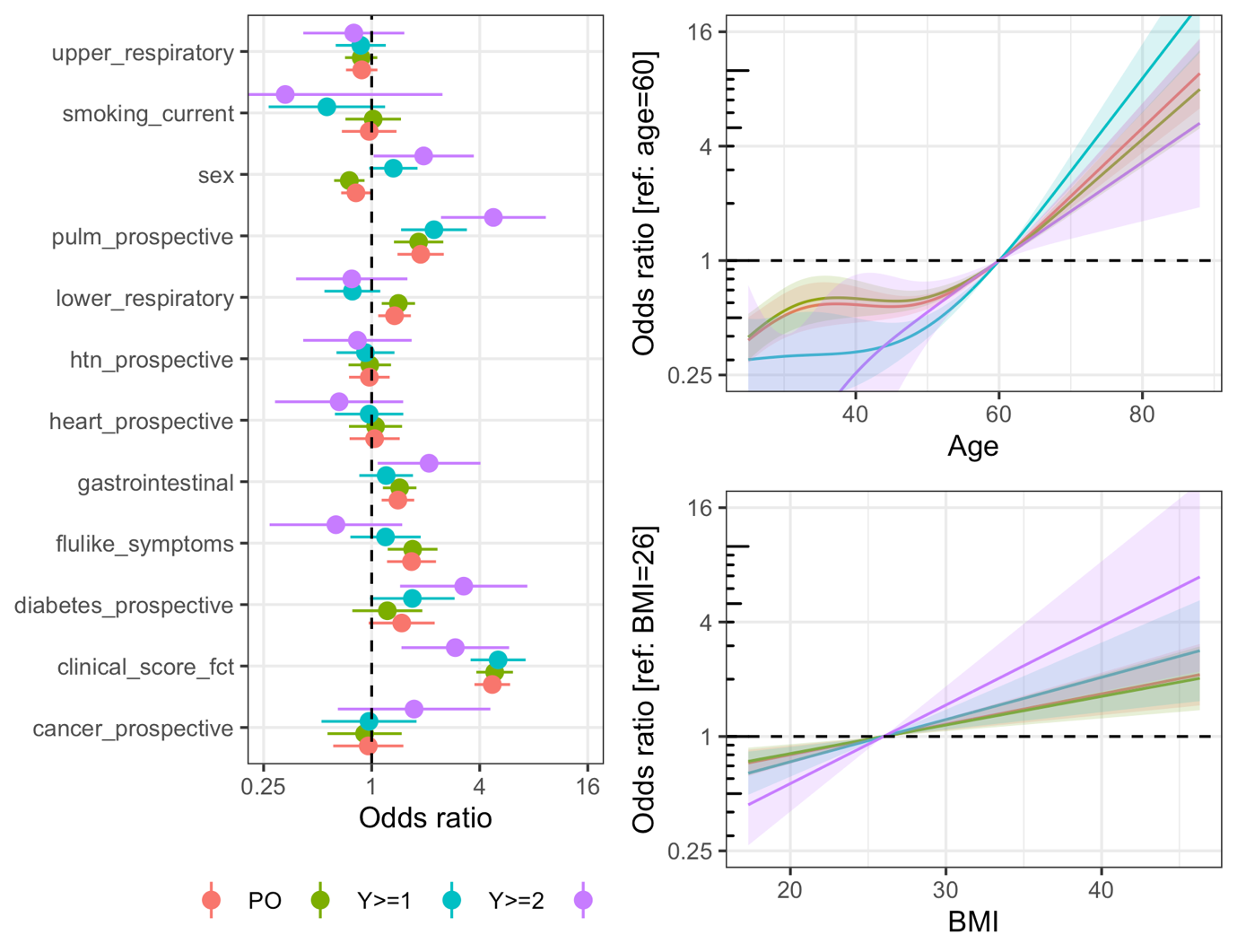


**Supplementary Figure 8.** A comparison of the beta coefficients (shown as odds ratios [OR]) of the fully specified proportional odds (PO) model and binary models of each outcome.


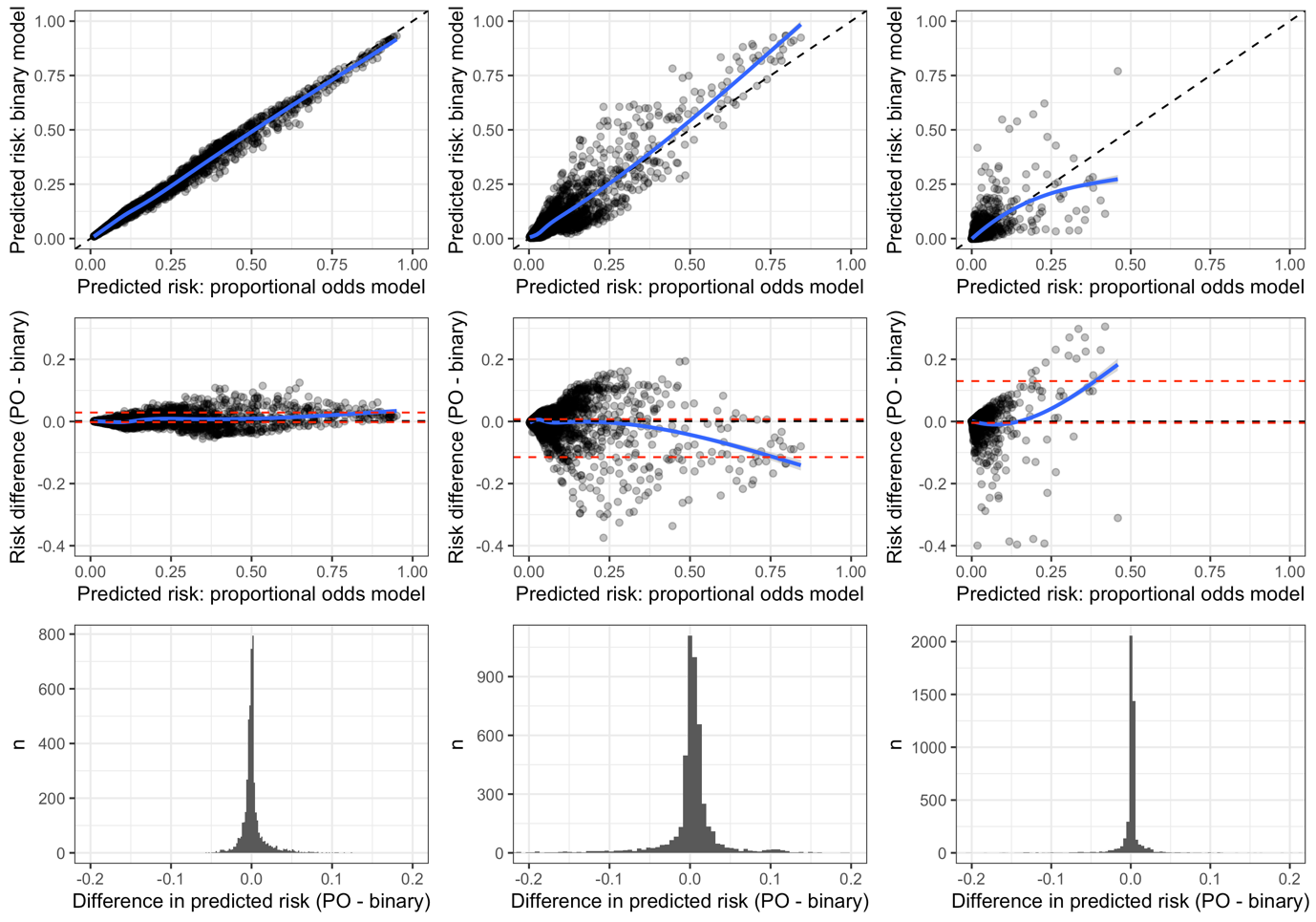


**Supplementary Figure 9.** A comparison of the predictions made by the fully specified proportional odds (PO) model and binary models of each outcome. The leftmost column of figures represents the outcome of clinical deterioration requiring urgent in-person evaluation at the LUH COVID-19 Outpatient Clinic, but not subsequent hospitalization, the middle column represents hospitalization and the rightmost column admission to intensive care unit (ICU) or death. The top row of figures shows the relationship between predicted risk of the PO model (X-axis) and the binary model (Y-axis) with the weighted scatterplot smoothing (LOWESS) relationships depicted as a blue line. A perfect relationship is shown as a diagonal dashed line. The middle row shows the risk difference between PO and binary models (Y-axis) as a function of predicted risk by the PO model (X-axis). The LOWESS risk difference is shown as a blue line. The minimum and maximum LOWESS risk difference is shown with dashed red lines. Finally, the bottom row depicts histograms of the risk difference. There is a slight tendency of the PO model to underpredict hospitalizations compared to the binary model for predictions above 50%. Similarly, there is a tendency for the PO model to overpredict ICU admissions or death for risks above 20%.

*
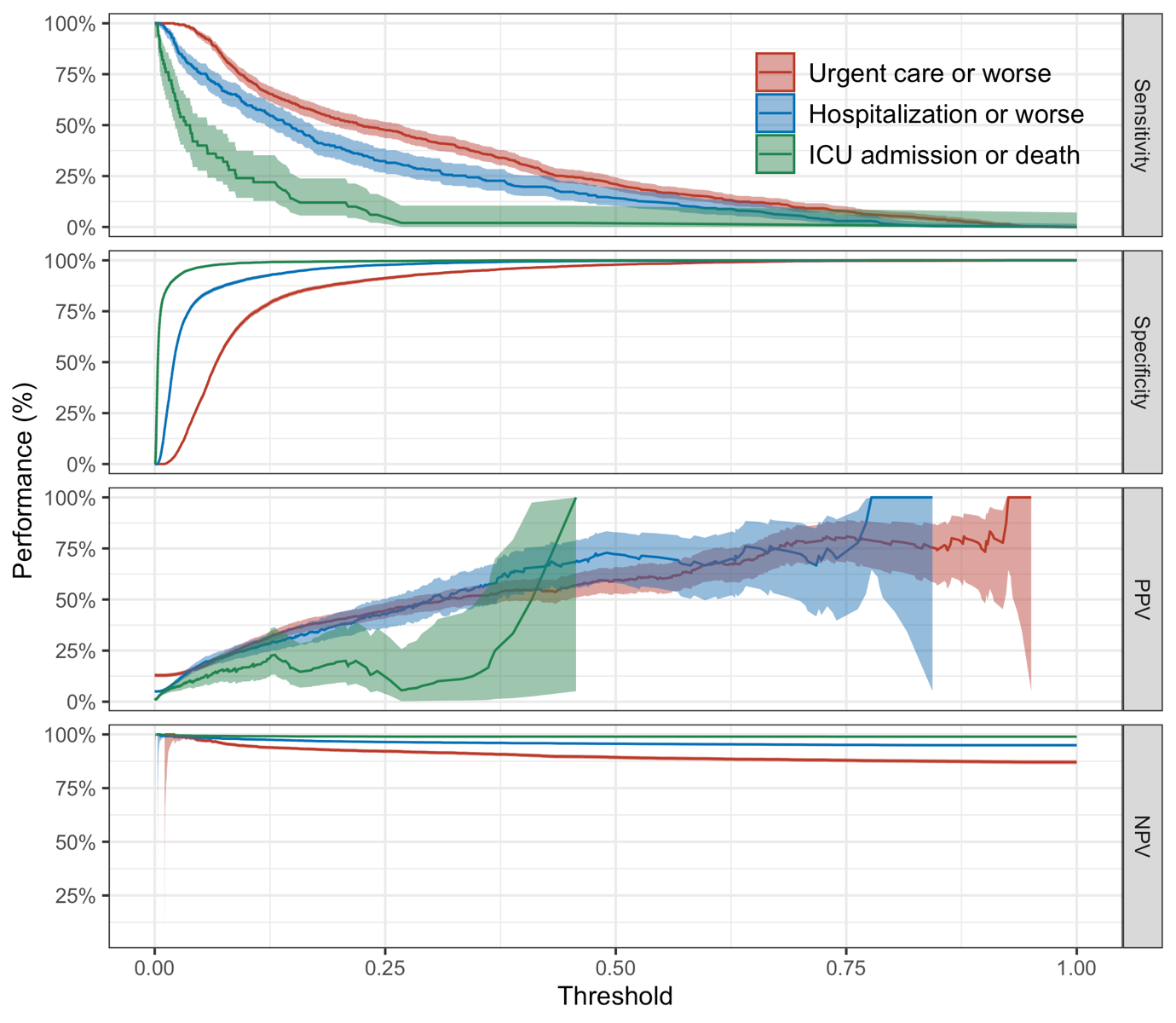
*

**Supplementary Figure 10.** The sensitivity, specificity, positive predictive value (PPV) and negative predictive value (NPV) of the prognostic model in the derivation cohort for the three outcomes. The x-axis shows the threshold of predicted probability of the outcome at which the performance is measured


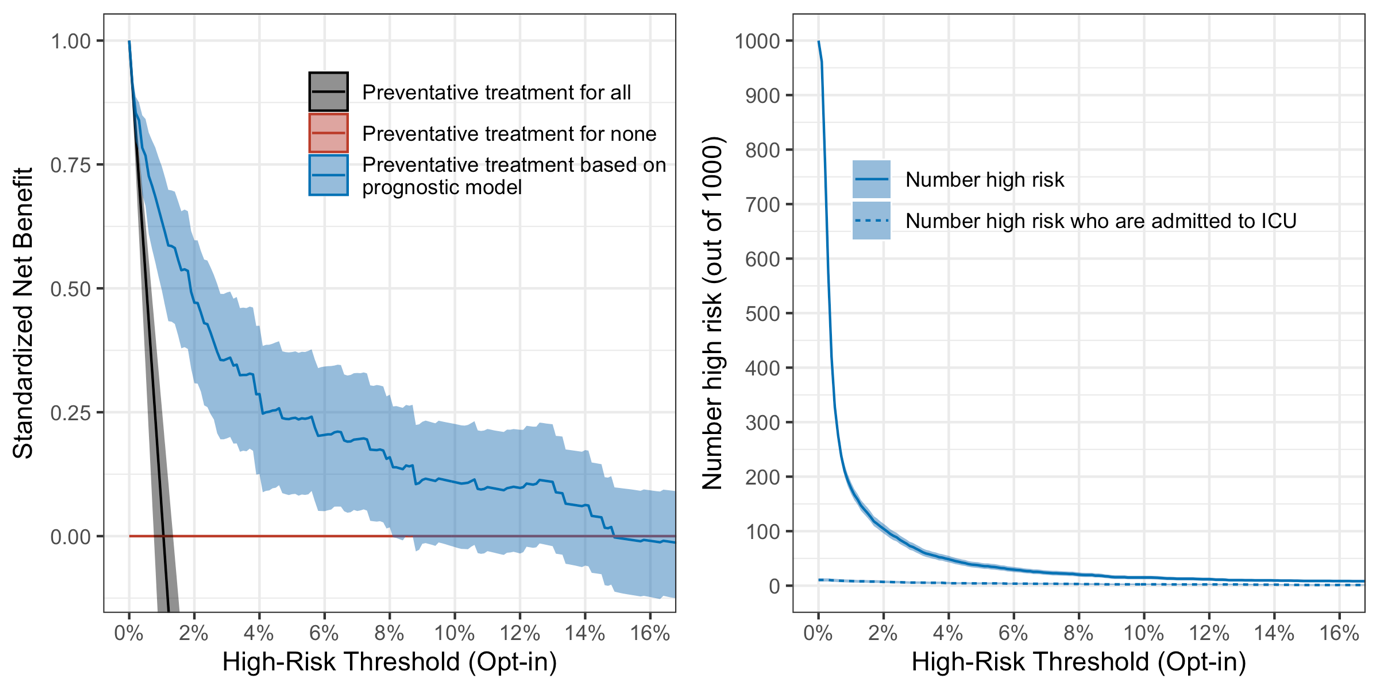


**Supplementary Figure 11.** The standardized net benefit of the prognostic model (blue line) compared with the strategies of treating all individuals (black line) and treating none (red line) over a range of risk thresholds. Panel A depicts the use of the prognostic model to offer patients who are at high-risk of ICU admission or death therapeutic intervention. The Y-axis represents the net increase in the proportion of high-risk patients who are offered treatment (out of a hypothetical maximum achieved when the true positive rate is one and the false positive rate is zero) compared with the strategy of treating no patients. Panel B shows the expected number of patients (out of 1000) who would be offered treatment (blue line) as a function of high-risk threshold. Also shown is the expected number of high-risk patients (blue dashed line) who later are admitted to ICU or died.


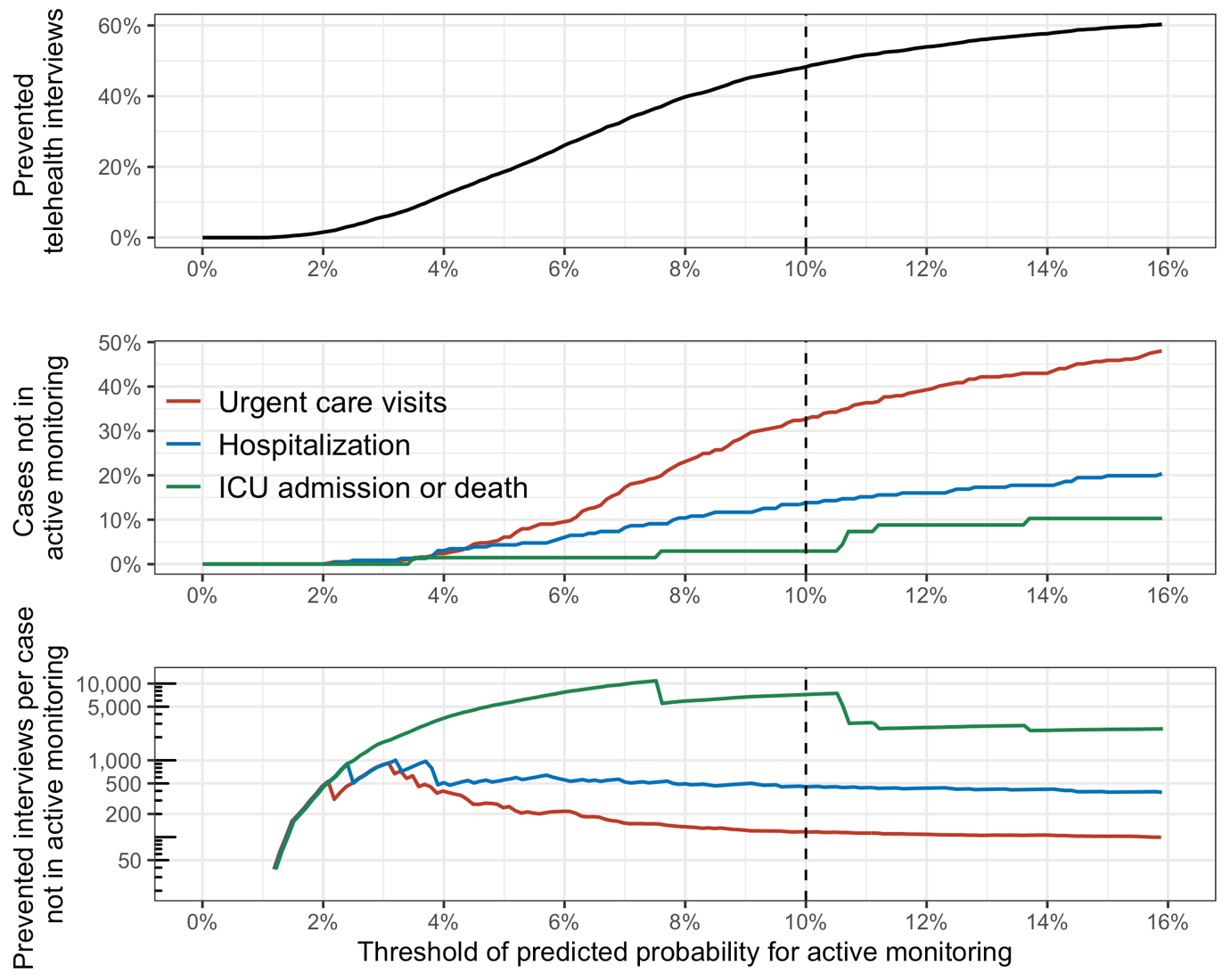


**Supplementary Figure 11.** Potential benefits and harms of implementing the prognostic model to triage into a telehealth service, using different thresholds for the predicted probability of the need for an urgent care visit or worse. The top panel illustrates the proportion of telehealth interviews that would have been avoided if those who had a predicted probability below the given threshold received only two (enrollment and discharge) interviews. The middle panel depicts the proportion of individuals who required escalation of care that would have received fewer interviews using the corresponding threshold. The bottom panel illustrates the ratio between these potential benefits and harms.
